# Long-term ovarian function risks associated with combined oral contraceptive administration in polycystic ovary syndrome: multisource data integration and protective mechanisms of metformin

**DOI:** 10.1186/s12967-026-08429-y

**Published:** 2026-06-15

**Authors:** Jingjing Li, Jiajia Wei, Chong Liu, Ping He, Linggang Zhou, Saiqiong Chen, Zeying Feng

**Affiliations:** 1 Department of Gynecology, Liuzhou Maternity and Child Healthcare Hospital, No. 50, Yingshan Avenue, ChengZhong District, Liuzhou, Guangxi 545001 China; 2 Liuzhou Hospital of Guangzhou Women and Children’s Medical Center, Liuzhou, Guangxi 545000 China; 3 Liuzhou Key Laboratory for Diagnosis and Treatment of Infertility related to Polycystic Ovary Syndrome, Liuzhou, China; 4 Xiangya School of Pharmaceutical Sciences, Central South University, Changsha, Hunan 410013 China; 5 Department of Obstetrics and Gynecology, The Fourth Affiliated Hospital of Guangxi Medical University, Liuzhou, Guangxi China; 6 Clinical Trial Institution Office, Liuzhou Hospital of Guangzhou Women and Children’s Medical Center, No. 50 Boyuan Avenue, Yufeng District, Liuzhou, Guangxi China

**Keywords:** Polycystic ovary syndrome, Combined oral contraceptives, Ovarian function risk, Multisource data, Metformin

## Abstract

**Background:**

Polycystic ovarian syndrome (PCOS) is a prevalent endocrine disorder in women of reproductive age. Furthermore, the potential risks of long-term use of combined oral contraceptives (COCs) on ovarian reserve function remain unclear. We aimed to assess the association systematically between COC use and ovarian function decline in patients with PCOS and to explore potential mechanisms associated with metformin.

**Methods:**

We analyzed 336 patients with PCOS and evaluated the association between COC use and ovarian function decline. A disproportionality analysis was performed to assess 3,424 COC-related ovarian-function adverse event reports in FAERS. Datasets of potential targets for ovarian function risk were constructed, and ovarian toxicity-related targets were screened. Intersection genes from the datasets were used to construct a protein–protein interaction (PPI) network. We identified core targets and pathways and predicted drug-target co-folded structures, while molecular dynamics simulations evaluated the binding stability and explored the potential regulatory effects of metformin.

**Results:**

Prolonged COC use was significantly associated with an increased risk of premature ovarian insufficiency and premature ovarian failure. A history of metformin use was associated with lower odds of the broader ovarian function decline outcome. Computational analyses suggested that COCs may be linked to ovarian toxicity-related pathways, whereas metformin may modulate these predicted interactions through competitive binding and conformational alterations.

**Conclusions:**

This study represents the first integration of multisource data to systematically characterize potential ovarian-function risks associated with long-term COC therapy in patients with PCOS and to explore putative mechanisms that may underlie these associations. The findings suggest that a history of metformin use may be associated with attenuated risks, but future prospective or randomized investigations are warranted to validate these clinical associations and hypothesized mechanisms, thereby optimizing individualized management of PCOS.

**Supplementary Information:**

The online version contains supplementary material available at 10.1186/s12967-026-08429-y.

## Introduction

Polycystic ovary syndrome (PCOS) is one of the most common endocrine disorders in women of reproductive age, affecting about 10–13% of this population [1, 2]. It is characterized by hyperandrogenism, ovulatory dysfunction, and polycystic ovarian morphology and is associated with infertility and multiple metabolic comorbidities [3, 4]. Current first-line management combines lifestyle intervention with pharmacotherapy, and COCs are widely prescribed to regulate menstrual cycles and relieve hyperandrogenic symptoms such as hirsutism and acne [5]. However, the long-term reproductive safety of COCs in women with PCOS has not been fully clarified. Premature ovarian insufficiency (POI), defined as loss of ovarian function before 40 years of age with elevated gonadotropins and low estradiol [6], occurs more than eight times as often in women with PCOS as in the general population [7], making it essential to understand whether COCs exposure further compromises ovarian reserve in this high-risk group.

The traditional view is that COCs exert their contraceptive and therapeutic effects by suppressing the hypothalamic–pituitary–ovarian axis, with their influence on ovarian function considered “completely reversible” following discontinuation. However, this viewpoint is grounded in studies involving healthy contraceptive users who typically exhibit a rapid restoration of ovulatory function and fertility after cessation [8–11]. Ovarian physiology in patients with PCOS differs from that in healthy women, manifesting as aberrant follicular development and a potential compromise in oocyte quality [12, 13]. These patients inherently constitute a high-risk demographic for diminished ovarian reserve (DOR) or even primary ovarian insufficiency (POI), sharing risk factors with these conditions, including autoimmune diseases, genetic predispositions, and environmental influences [14, 15]. Therefore, long-term exposure to exogenous sex hormones may cumulatively affect the inherently vulnerable ovarian environment in patients with PCOS, leading to irreversible consequences that differ from those observed in healthy populations. These conditions may elevate the risk of a secondary profound decline in ovarian function, such as POI. However, extant safety investigations predominantly emphasize the short-term ramifications of COCs (e.g., thrombotic and depressive risks) [16, 17], with a conspicuous paucity of evidence concerning ovarian safety during protracted use.

Metformin is widely used to treat insulin resistance and metabolic dysfunction in PCOS and also exerts direct ovarian actions. Experimental data show that metformin activates AMP-activated protein kinase, attenuates NF-κB–dependent inflammasome activity, modulates the miR-670-3p/NOX2/ROS axis, improves ovarian angiogenesis and follicular development, and protects the ovarian reserve from oxidative stress–induced injury and granulosa-cell pyroptosis [18–21]. In clinical practice, metformin is frequently prescribed concomitantly with COCs to address both metabolic and reproductive manifestations of PCOS. Randomized trials and systematic reviews comparing COCs alone with COCs plus metformin indicate that adding metformin can mitigate some adverse metabolic and vascular effects of COCs and improve menstrual regularity and biochemical hyperandrogenism [22, 23]. These findings suggest that metformin may partly counteract COCs-related metabolic and inflammatory stress, but existing studies have focused on short-term cardiometabolic and reproductive outcomes and have not directly examined COCs-related DOR, POI, or long-term ovarian function decline.

Based on these considerations, we hypothesized that prolonged COCs use in women with PCOS is associated with an increased risk of ovarian function decline and that a documented history of metformin use may mitigate this risk by modulating inflammatory and oxidative pathways. To test this hypothesis, we integrated real-world data from a retrospective PCOS cohort, pharmacovigilance data from the U.S. Food and Drug Administration (FDA) Adverse Event Reporting System (FAERS), and network toxicology, molecular docking, and molecular dynamics (MD) simulations. This multi-stage approach allows us to progressively build evidence from clinical observations to mechanistic insights, as outlined in the following sections. Our objectives were to (i) quantify the association between long-term COCs exposure and ovarian function decline in PCOS, (ii) assess the potential protective association of metformin use, and (iii) explore putative molecular mechanisms potentially associated with COC-related ovarian function signals and metformin-associated modulation.

## Methods

**Fig. 1:**
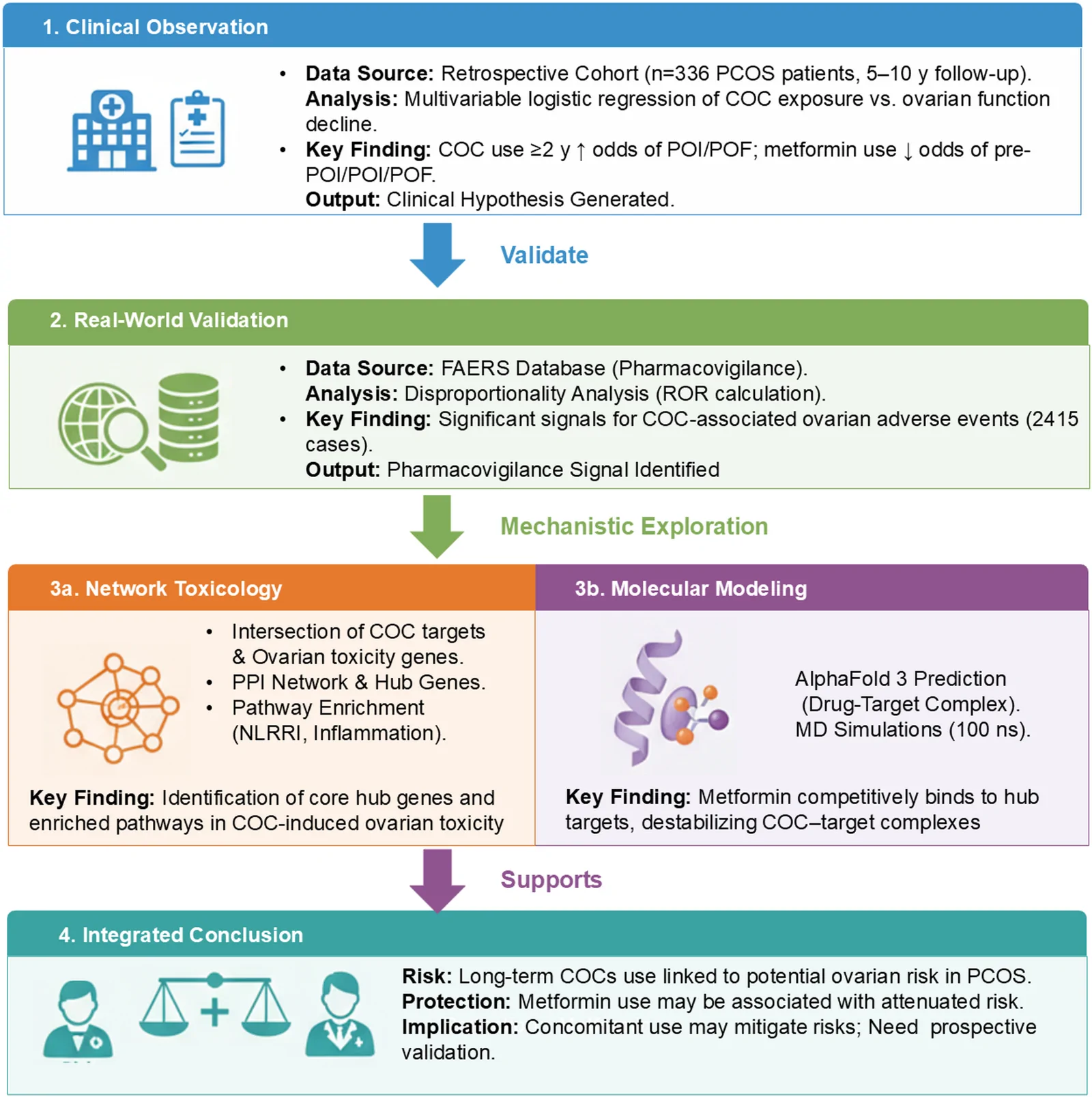
Integrated workflow illustrating the connection between clinical data analysis, pharmacovigilance assessment, and computational modeling

Our study followed a multi-stage, integrated design as outlined in Fig. 1. To ensure logical progression, the workflow begins with clinical data to generate hypotheses, followed by pharmacovigilance signal assessment, and culminates in mechanistic exploration. The workflow began with a retrospective clinical analysis to quantify the potential risk of long-term COCs use in PCOS patients and to generate an initial clinical hypothesis regarding both COCs risks and the potential protective associations of metformin use. This was followed by a pharmacovigilance study using the FAERS database to examine whether similar ovarian-function-related reporting signals could be observed in a large spontaneous reporting database. Finally, to elucidate the potential biological basis for these observations, we conducted an in silico mechanistic investigation using network toxicology and molecular modeling to explore the underlying pathways and potential metformin-associated modulatory mechanisms.

### Retrospective analysis in patients with PCOS

This was an observational retrospective cohort study based on real-world data from our dedicated PCOS database at Liuzhou Hospital of Guangzhou Women and Children’s Medical Center, formerly the East Campus of Liuzhou Maternity and Child Healthcare Hospital. The baseline variables include age, menstrual history, body mass index (BMI), hormonal profiles (follicle-stimulating hormone [FSH], luteinizing hormone [LH], estradiol, and testosterone [T]), anti-Müllerian hormone (AMH) levels, treatment modalities, encompassing both COC regimens and a history of metformin use, and smoking history. Within this retrospective framework, metformin use was specifically analyzed as a documented exposure history; thus, findings reflect observed clinical associations rather than prospective intervention effects. We aimed to assess long-term (5–10 years) ovarian function decline in early-diagnosed PCOS patients, focusing on COCs’ influence. Patients diagnosed between 2015 and 2018 were selected for comprehensive baseline data and adequate follow-up. The study was conducted in accordance with the ethical principles of the Declaration of Helsinki and its later amendments. The Ethics Committee of Liuzhou Hospital of Guangzhou Women and Children’s Medical Center approved this study (Approval No.: Quick Review-Research-2024-232), and written informed consent was obtained from all participants.

Inclusion criteria were as follows: (1) Confirmed PCOS diagnosis per Rotterdam criteria; (2) Age at diagnosis: 15–35 years; (3) Comprehensive baseline data and 5–10 years of follow-up. Exclusion criteria included: (1) PCOS-mimicking conditions (e.g., hyperprolactinemia, Cushing’s syndrome, congenital adrenal hyperplasia, androgen-secreting tumors, active thyroid disease); (2) Disorders affecting ovarian reserve (e.g., smoking history, genetic diseases, endometriosis, autoimmune diseases, chromosomal abnormalities); (3) Surgical history (ovarian/fallopian tube resection) or chemotherapy/radiotherapy; (4) Use of non-COCs hormonal contraceptives (e.g., progestin-only products including pills, implants, levonorgestrel IUD, or contraceptive vaginal rings), to focus on COCs vs. non-hormonal methods; (5) Recent hormonal use (within 6 months of assessment).

To minimize confounding in the final cohort of 336 patients, we excluded comorbidities directly affecting ovarian reserve and adjusted for key metabolic factors (diabetes, insulin resistance) and calendar time in multivariable models. BMI at diagnosis was used as a proxy for metabolic and lifestyle factors.

Participant contraceptive methods were categorized as COCs users (ethinyl estradiol plus progestin) or non-users (no hormonal contraception) [11]. Ovarian function among all participants was categorized into normal ovarian reserve (NOR), prodromal phase of POI (pre-POI), POI, and premature ovarian failure (POF) groups based on recent FSH levels and menstrual status in patients with PCOS. Individuals with normal endocrine hormone profiles (5 IU/L < FSH < 10 IU/L and AMH > 1.2 ng/mL) were assigned to the NOR group. Diagnostic criteria for conditions associated with DOR were as follows. Women in the pre-POI presenting with regular or irregular menstruation accompanied by elevated FSH levels (10 IU/L < FSH ≤ 25 IU/L, evaluated on two occasions with an interval of at least 4 weeks). POI diagnostic criteria included oligomenorrhea/amenorrhea persisting for at least 4 months and elevated FSH levels > 25 IU/L (evaluated on two occasions with an interval of at least 4 weeks), wherein 25 IU/L < FSH ≤ 40 IU/L was defined as early POI, and FSH > 40 IU/L was defined as POF [24]. Ovarian function decline is a continuous and progressive process. To address the heterogeneity of this condition and improve the representativeness and generalizability of the study findings, ovarian function decline was defined in the primary analysis as early POI and POF (Outcome 1); in the secondary analysis, this scenario encompassed pre-POI, early POI, and POF (Outcome 2).

### Pharmacovigilance data analysis

We conducted a pharmacovigilance study to examine the adverse drug reactions associated with DOR linked to COCs, as reported in the FAERS database. The database serves as a public repository for safety reports submitted by patients, healthcare professionals, and pharmaceutical companies, with quarterly data updates [25–27]. We retrieved the FAERS data files encompassing the period from quarter 4 of 2014 to quarter 3 of 2024. Prior to analysis, a data cleaning process was implemented. To address duplicate entries, we followed the FDA’s recommended methodology by retaining only the most recent report (based on CASEID) for cases with identical patient demographics, event dates, and reported drugs. Reports with missing data for drug names or adverse event terms were also excluded to ensure the quality of the analysis. We employed both generic and brand names to identify short-acting COCs, encompassing combinations such as cyproterone and ethinylestradiol (EE), drospirenone (DRSP) and EE, desogestrel and EE, levonorgestrel and EE and norethisterone and EE. Only cases of DOR suspected of being attributable to COCs were included; those in which COCs were documented as concomitant medications were excluded from the analysis.

Adverse events (AEs) were classified according to the guidelines outlined in the Medical Dictionary for Regulatory Activities (MedDRA version 25.1) [28]. Preferred terms (PTs) were used to define adverse reactions associated with DOR, including ovarian failure (OF), premature menopause (PM), ovarian dysfunction (OD), increased follicular atresia (IFA), DOR, amenorrhea, ovarian disorder, ovarian atrophy, and low antral follicle count.

### Network toxicology analysis

Network toxicology investigates potential toxicity mechanisms by constructing multi-component regulatory networks and identifying toxic targets, thereby elucidating the molecular basis of toxicity [29, 30]. Artificial intelligence-based AlphaFold 3 predictions of target-drug binding structures have emerged as an effective tool utilized to study drug-target interaction relationships, enabling accurate forecasting of interactions between small molecules (ligands) and biomacromolecules (such as proteins, DNA, or RNA, termed receptors). This approach plays a pivotal role in exploring drug targets, screening potential therapeutic compounds, and optimizing lead compounds [31]. MD simulations offer an intuitive visualization of the dynamic interaction processes between drugs and targets, effectively demonstrating the binding stability between drugs and receptors. This methodology has been extensively employed to address challenges that are difficult to resolve through experimental approaches alone, such as the pathogenic mechanisms of diseases induced by amyloid protein misfolding, virtual screening, drug-target interactions, and resistance mechanisms resulting from target mutations [32].

We utilized network toxicology to analyze the toxic effects of estrogenic compounds on the ovaries while concurrently examining the modulatory effects of metformin on adverse reactions induced by combinations of estrogen and progestin agents. Furthermore, by employing artificial intelligence-based co-folding structure prediction and MD simulation techniques, we preliminarily evaluated predicted key components and hub targets implicated in ovarian toxicity elicited by estrogenic compounds and estrogen–progestin combinations, as well as the regulatory influence of metformin on these adverse reactions.

#### Prediction of potential COC-related toxicity targets

The establishment of a computer-readable small-molecule library for estrogenic compounds and estrogen–progestin combinations was performed using the PubChem database (https://pubchem.ncbi.nlm.nih.gov/) and the ChEMBL database (https://www.ebi.ac.uk/chembl/). Datasets derived from PubChem exhibit a broader distribution within the chemical space of approved drugs, whereas those from ChEMBL are more concentrated in the densest regions of the approved drug chemical space [33]. The constructed small-molecule library, incorporating SMILES structures and other pertinent key information, was subsequently employed with the SwissTargetPrediction database (http://swisstargetprediction.ch/), the DrugBank database (https://www.drugbank.ca/), ChEMBL, and analogous repositories to predict drug-related targets.

#### Retrieval of ovarian toxicity targets

Five keywords—OF, PM, OD, IFA, and DOR—were utilized to gather relevant targets. To ensure a systematic and comprehensive collection of targets associated with adverse reactions, the aforementioned adverse reaction-related targets were searched across multiple databases, followed by integrated analysis of the results. Pertinent targets were derived from databases including GeneCards (https://www.genecards.org/), OMIM (https://omim.org/), and DisGeNET (https://disgenet.com/).

#### Relevant targets between COCs and adverse reactions

Using the online tool Venny 2.1.0 (https://bioinfogp.cnb.csic.es/tools/venny/), the estimated COCs targets were compared to gene targets linked with ovarian function decline. The intersections of these datasets identified possible targets through which COCs may exert harmful effects on ovarian function, and Venn diagrams were created to illustrate the overlaps.

#### Construction of PPI networks

To identify the interactions between targets associated with ovarian function decrease, the intersection targets were entered into the STRING database (https://string-db.org/) to create a PPI network. Drug components were then connected to their predicted targets to form an extended drug-drug combination-target association network. The generated networks were loaded into Cytoscape 3.10.3 for visualization, with further clustering analysis of important modules performed using the CentiScape 2.2 plugin and Molecular Complex Detection (MCODE) techniques. This facilitated the identification of core targets pertinent to the AEs.

#### Pathway enrichment analysis of core targets for estrogen–progestin adverse effects

Metascape [34] (http://metascape.org/gp/index.html) integrates over 40 bioinformatics databases, furnishing comprehensive tools for biological pathway enrichment analysis, PPI network analysis, and extensive gene annotation. The Kyoto Encyclopedia of Genes and Genomes (KEGG) pathway enrichment database amalgamates genomic, chemical, and system-level functional data and establishes connections between genetic information and biological functions, thereby illuminating the genetic and biochemical foundations of life processes. Using the Metascape database, pathway enrichment analysis of adverse reactions was performed on core targets to delineate the key signaling pathways involved in COCs-induced toxicity and to probe their underlying mechanisms.

#### Drug-target binding co-folded structures based on AlphaFold 3

The local installation of AlphaFold 3.0.1 facilitates accurate predictions of interaction structures between individual or multiple drugs and their respective targets, serving as a valuable resource for exploring drug-target interactions and the dynamics involving various drugs and targets. Five independent complex structure predictions were generated for each protein–dual-ligand system, and the model with the highest confidence was chosen for subsequent molecular dynamics simulations. COCs and metformin were utilized as ligands for structural prediction. The primary targets associated with adverse reactions were chosen from the PPI network to function as receptors. The three-dimensional structures of COCs were retrieved from the PubChem database and stored in SDF format, after which SMILES structures were extracted. Amino acid residue sequences of the core toxicity were retrieved from the UniProt database (www.uniprot.org) using target names, ensuring that the sequences were specific to humans. AlphaFold 3 was employed to predict the co-folded structures of various targets with estrogens, progestins, and their combinations with metformin at the core targets. The predictive outcomes were visualized with ChimeraX and analyzed using MOE software. The results included co-crystallized structures, drug-target interaction relationships, and similar features.

#### MD simulations of drug–target binding dynamics and stability

To investigate the competitive binding behavior of estrogen and progestin, respectively, in combination with metformin as dual ligands in the binding pockets of target proteins, as well as their impacts on binding stability, MD simulations were performed on the complex systems using the AMBER simulation software (AMBER 24). Specifically, to elucidate the effects of metformin binding on the relevant drug combinations involving estrogen and progestin, the two small-molecule ligands were parameterized separately using the antechamber tool, applying the GAFF2 force field and assigning charges via the AM1-BCC or Gasteiger method. Meanwhile, proteins were modeled using the ff14SB force field, and the complexes were solvated with the TIP3P water model, incorporating a 10 Å solvent buffer and adding Na⁺ or Cl⁻ ions to neutralize the total charge. The complex structures, derived from AlphaFold 3 predictions and comprising the protein along with two ligand molecules, underwent a systematic simulation process after system construction: this included energy minimization, heating, equilibration, and the production MD phase, during which a 100 ns simulation was conducted under NPT conditions at 300 K and 1 atm, with a 2 fs timestep and hydrogen bonds constrained using the SHAKE algorithm. Furthermore, temperature regulation was achieved using a Langevin thermostat with trajectory, and energy information recorded every 10 ps. Upon completion of the simulations, the CPPTRAJ tool was employed to analyze the trajectory files, encompassing the root-mean-square deviation (RMSD) of the overall protein and the two ligands, root-mean-square fluctuation (RMSF) of key residues in the binding pocket, changes in inter-ligand distances, formation and duration of key hydrogen bonds, as well as spatial repulsion relationships between the two ligands. Consequently, these analyses reveal the dynamic interaction patterns of ligand A and ligand B within the binding pocket, thereby assessing the presence of competitive binding or synergistic stabilization effects and providing a theoretical basis for elucidating multi-ligand regulatory mechanisms.

### Statistical analysis

Continuous variables are presented as mean ± standard deviation or median (interquartile range [IQR]) and compared using independent t-tests or Mann–Whitney U tests, as appropriate. Categorical variables are expressed as n (%) and analyzed using the chi-square test or Fisher’s exact test. Regression models were used to assess the association between COCs use and the risk of ovarian function decline. Univariate logistic regression analysis was applied for preliminary variable screening, followed by multivariable logistic regression to adjust for confounding factors, including calendar time, diabetes, and insulin resistance, to determine the relationship between COCs therapy and the risk of ovarian function decline. Furthermore, to comprehensively evaluate ovarian function decline, two distinct outcome definitions were utilized. Logistic regression and descriptive analyses were performed using SPSS version 26, whereas multiple imputation was performed using R software version 4.5.3, and statistical significance was defined as *P* < 0.05.

Missing data were limited in the cohort, primarily involving baseline height and weight (3.6%), testosterone (2.4%), and anti-Müllerian hormone (2.1%). Among COC users, duration of discontinuation was missing in 4.2% of cases. Body mass index was derived from height and weight and was therefore handled through passive derivation rather than treated as an independent missing variable. Little’s MCAR test did not reject the MCAR assumption (detailed missingness profiles and test results are provided in Supplementary Table S2). Missing covariate data were handled using multiple imputation under the MAR framework with predictive mean matching in R software version 4.5.3. Five imputed datasets were generated over 10 iterations. Because multiple imputation accounts for missing covariate data while preserving the analytic sample under the MAR assumption, pooled estimates from the imputed datasets were used as the primary regression results. Available-data analyses before imputation were retained as supplementary analyses for transparency (Supplementary Tables S3 and S4).

Disproportionality analysis is a fundamental data mining and signal detection technique in the domains of pharmacovigilance and pharmacoepidemiologic research, with the objective of identifying statistically significant safety signal associations between drugs and AEs within spontaneous reporting databases of adverse drug reactions. To perform the disproportionality analysis [35], we used the reporting odds ratio (ROR), a metric computed as the ratio of the odds of reporting DOR events for the target drug divided by the corresponding odds for all other drugs in the database during the same period (the comparator group) [36]. This comparator group was used consistently for all ROR computations, including the overall analysis for COCs and the subgroup analyses for individual agents. Significant signals were defined as those exhibiting a lower bound of the 95% confidence interval (CI) for ROR > 1, accompanied by a minimum of three reported cases [37].

## Results

### COCs use and risk of ovarian function decline

We conducted a retrospective analysis to ascertain whether ovarian function decline manifests during long-term follow-up (5–10 years) among 342 women of reproductive age with an early diagnosis of PCOS. After exclusions, the final study population comprised 336 women, including COCs users (*n* = 167) and non-users (*n* = 169) (Table 1). Among patients with PCOS, 14.3% developed secondary ovarian function decline, with an incidence rate of 20.4% observed among COCs users. Based on the multiple imputation datasets, regression models were used to evaluate the association between COCs use, metformin use, and the risk of decline in ovarian function. We observed that prolonged use of COCs (≥ 2 years) in patients with PCOS was significantly associated with an increased risk of severe ovarian function decline (Outcome 1: POI + POF) across all models, including the fully adjusted Model 2 (adjusted OR 7.588, 95% CI: 1.818–31.662, *P* = 0.006). However, for the broader definition of ovarian function decline (Outcome 2: pre-POI, POI, and POF), prolonged COC use was only significant in the crude analysis (OR 3.188, 95% CI: 1.287–7.896, *P* = 0.012) and Model 1 (OR 4.048, 95% CI: 1.561–10.499, *P* = 0.004); this association was attenuated and no longer statistically significant after full adjustment for covariates in Model 2 (adjusted OR 2.424, 95% CI: 0.865–6.794, *P* = 0.092) (Table 2).

**Table 1:** Baseline characteristics of patients with PCOS stratified by COC use

Variable	COC users (*n* = 167)	Non-COC users (*n* = 169)	*P* value
Age at menarche, years, median (IQR)	13 (12–14)	12 (12–13)	0.062
Age at PCOS diagnosis, years, median (IQR)	24 (21–26)	24 (21–27)	0.625
BMI at PCOS diagnosis, kg/m², median (IQR)	26.4 (23.5–28.3)	25.0 (23.0–26.8)	< 0.001
FSH at PCOS diagnosis, mIU/mL, mean (SD)	5.5 (1.0)	5.6 (1.0)	0.127
Testosterone at PCOS diagnosis, ng/dL, median (IQR)	17.3 (13.2–52.4)	16.6 (13.0–32.6)	0.116
AMH at PCOS diagnosis, ng/mL, median (IQR)	8.6 (5.5–11.5)	7.4 (5.4–11.4)	0.737
DOR, n (%)			0.002
Present	34 (20.4)	14 (8.3)	
Absent	133 (79.6)	155 (91.7)	
DOR type, n (%)			0.779
pre-POI	23 (67.6)	9 (64.3)	
POI	7 (20.6)	4 (28.6)	
POF	4 (11.8)	1 (7.1)	
Documented metformin use, n (%)	77 (46.1)	43 (25.4)	< 0.001

Note: Data are presented as mean (SD), median (interquartile range [IQR]), or n (%), as appropriate. Baseline characteristics are summarized using available observed data. Missingness was low and was handled in regression analyses as described in the Statistical analysis section. Percentages for DOR type were calculated among participants with DOR. AMH, anti-Müllerian hormone; BMI, body mass index; COC, combined oral contraceptive; DOR, diminished ovarian reserve; FSH, follicle-stimulating hormone; PCOS, polycystic ovary syndrome; POI, premature ovarian insufficiency; POF, premature ovarian failure

**Table 2:** Pooled multiple-imputation logistic regression models for ovarian function decline in patients with PCOS

Variable	Crude OR (95% CI)	*P* value	Model 1 OR (95% CI)	*P* value	Model 2 OR (95% CI)	*P* value
**Outcome 1: POI + POF**						
Age at PCOS diagnosis, years	0.975 (0.869, 1.093)	0.658	0.955 (0.846, 1.079)	0.462	0.938 (0.795, 1.105)	0.440
Age at COC initiation, years#	1.035 (0.897, 1.193)	0.639	1.051 (0.892, 1.238)	0.551	1.053 (0.891, 1.244)	0.545
BMI ≥ 25 kg/m² vs. < 25 kg/m²	1.067 (0.359, 3.170)	0.907	1.358 (0.434, 4.252)	0.599	1.399 (0.353, 5.538)	0.631
Long-term COC use ≥ 2 years vs. < 2 years	6.818 (2.150, 21.623)	0.001	10.083 (2.876, 35.341)	< 0.001	7.588 (1.818, 31.662)	0.006
COC discontinuation ≥ 12 months vs. < 12 months#	0.544 (0.149, 1.983)	0.354	0.491 (0.116, 2.084)	0.333	0.535 (0.123, 2.321)	0.401
Documented metformin use vs. no documented use	0.400 (0.111, 1.441)	0.161	0.266 (0.068, 1.048)	0.058	0.184 (0.031, 1.105)	0.064
**Outcome 2: pre-POI + POI + POF**						
Age at PCOS diagnosis, years	0.964 (0.899, 1.034)	0.309	0.958 (0.891, 1.030)	0.242	0.942 (0.854, 1.039)	0.230
Age at COC initiation, years#	1.007 (0.923, 1.100)	0.868	1.007 (0.919, 1.104)	0.878	1.010 (0.920, 1.108)	0.836
BMI ≥ 25 kg/m² vs. < 25 kg/m²	1.621 (0.772, 3.403)	0.201	1.820 (0.843, 3.930)	0.127	1.828 (0.743, 4.500)	0.187
Long-term COC use ≥ 2 years vs. < 2 years	3.188 (1.287, 7.896)	0.012	4.048 (1.561, 10.499)	0.004	2.424 (0.865, 6.794)	0.092
COC discontinuation ≥ 12 months vs. < 12 months#	0.880 (0.369, 2.097)	0.771	0.863 (0.343, 2.172)	0.753	0.926 (0.362, 2.368)	0.871
Documented metformin use vs. no documented use	0.707 (0.362, 1.381)	0.309	0.569 (0.281, 1.149)	0.115	0.360 (0.139, 0.929)	0.035

Note: Data are presented as odds ratios (ORs) with 95% confidence intervals (CIs) and P values pooled across five imputed datasets. Outcome 1 was defined as POI + POF; Outcome 2 was defined as pre-POI + POI + POF. The primary full-cohort models included all 336 patients; Model 2 further adjusted for calendar time, diabetes, and insulin resistance. In the full-cohort analysis, the reference category for long-term COC use comprised non-users and COC users with cumulative exposure < 2 years. # Variables marked with # were applicable only to COC users and were analyzed in separate exploratory models restricted to COC users only (*n* = 167); they were not included as covariates in the primary full-cohort models. COC, combined oral contraceptive; PCOS, polycystic ovary syndrome; POI, premature ovarian insufficiency; POF, premature ovarian failure

Regarding documented metformin use, the analysis suggested a protective association that was most evident for the broader outcome definition. A history of metformin use was significantly associated with lower odds of the broad spectrum of ovarian function decline (Outcome 2) in the fully adjusted Model 2 (adjusted OR 0.360, 95% CI 0.139–0.929, *P* = 0.035). For the more severe outcome definition (Outcome 1: POI + POF), the association was directionally similar but did not reach formal statistical significance (Model 1: *P* = 0.058; Model 2: adjusted OR 0.184, 95% CI 0.031–1.105, *P* = 0.064). Available-data analyses before imputation showed broadly similar directions for the main associations and are provided for transparency in Supplementary Tables S3 and S4.

### COCs use and ovarian function decline in FAERS

Reports of ovarian-function-related AEs in which COCs were reported as suspected drugs involved 2,415 individuals. The clinical characteristics of the patients experiencing COCs-related ovarian function decline are shown in Table 3. Most ovarian-function-related AE reports occurred in individuals aged 18–30 years, accounting for 46.70%, followed by those aged 31–44 years (38.13%). The participants were predominantly consumers (76.45%), with contraception being the primary indication (67.37%). Levonorgestrel was the most prevalent (1,927 cases, 79.8%), followed by DRSP (424 cases, 17.6%). Other serious medical events (80.28%) were the most frequently reported severe outcomes, followed by hospitalization (13.70%) and disability (5.37%).

**Table 3:** Demographic characteristics of FAERS reports involving COC-related ovarian-function adverse events

Characteristic	All COCs	Levonorgestrel	Drospirenone	Desogestrel	Norethisterone	Cyproterone acetate
**Total cases**	2,415	1,927	424	26	19	19
**Age**						
Data available	1,319	1,238	35	15	14	17
< 18 years	59 (4.47%)	43 (3.47%)	3 (8.57%)	–	13 (92.86%)	–
18–30 years	616 (46.70%)	584 (47.17%)	20 (57.14%)	10 (66.67%)	–	2 (11.76%)
31–44 years	503 (38.13%)	477 (38.53%)	11 (31.43%)	3 (20.00%)	1 (7.14%)	11 (64.71%)
45–60 years	140 (10.61%)	133 (10.74%)	1 (2.86%)	2 (13.33%)	–	4 (23.53%)
> 61 years	1 (0.08%)	1 (0.08%)	–	–	–	–
**Indications**						
Data available	1,805	1,336	410	21	19	19
Contraception	1,216 (67.37%)	1,120 (83.83%)	79 (19.27%)	14 (66.67%)	–	3 (15.79%)
Menorrhagia	54 (2.99%)	53 (3.97%)	1 (0.24%)	–	–	–
Other indications	535 (29.64%)	163 (12.20%)	330 (80.49%)	7 (33.33%)	19 (100%)	16 (84.21%)
**Reporting region**						
Data available	2,415	1,927	424	26	19	19
United States	1,939 (80.29%)	1,533 (79.55%)	399 (94.10%)	5 (19.23%)	2 (10.53%)	–
Other countries	476 (19.71%)	394 (20.45%)	25 (5.90%)	21 (80.77%)	17 (89.47%)	19 (100%)
**Reporters**						
Data available	2,382	1,895	423	26	19	19
Consumer	1,821 (76.45%)	1,384 (73.03%)	410 (96.93%)	21 (80.77%)	3 (15.79%)	3 (15.79%)
Physician	305 (12.80%)	299 (15.78%)	5 (1.18%)	–	1 (5.26%)	–
Other health professional	120 (5.04%)	104 (5.49%)	3 (0.71%)	1 (3.85%)	12 (63.16%)	–
Unclassified healthcare provider	93 (3.90%)	79 (4.17%)	5 (1.18%)	1 (3.85%)	1 (5.26%)	7 (36.84%)
Pharmacist	28 (1.18%)	24 (1.27%)	–	2 (7.69%)	2 (10.53%)	–
Lawyer	15 (0.63%)	5 (0.26%)	–	1 (3.85%)	–	9 (47.37%)
**Outcomes**						
Data available	1,080	993	32	20	16	19
Other serious medical event	867 (80.28%)	820 (82.58%)	20 (62.50%)	11 (55.00%)	16 (100%)	–
Hospitalization	148 (13.70%)	128 (12.89%)	10 (31.25%)	6 (30.00%)	–	4 (21.05%)
Disability	58 (5.37%)	39 (3.93%)	2 (6.25%)	3 (15.00%)	–	14 (73.68%)
Life-threatening	3 (0.28%)	2 (0.20%)	–	–	–	1 (5.26%)
Other	4 (0.37%)	4 (0.40%)	–	–	–	–

Note: Data are presented as n (%) unless otherwise indicated. Percentages were calculated using the number of reports with available data for each characteristic as the denominator. Serious outcomes were categorized according to the primary reported serious outcome. COC, combined oral contraceptive; FAERS, FDA Adverse Event Reporting System

Our real-world study derived from the FAERS database encompassed 3,424 AEs associated with DOR linked to COCs, amenorrhea (*n* = 3,346), PM (*n* = 37), and ovarian disorder (*n* = 21), representing the top three most frequently reported PTs. We identified increased reporting odds for several ovarian-function-related PTs associated with COCs, including amenorrhea (ROR = 21.59 [95% CI: 20.66–22.57]), ovarian atrophy (ROR = 8.39 [95% CI: 3.67–19.17]), ovarian disorder (ROR = 2.26 [95% CI: 1.46–3.51]), and PM (ROR = 3.80 [95% CI: 2.72–5.31]). Furthermore, subgroup analyses for each agent revealed that levonorgestrel (ROR = 33.57) and DRSP (ROR = 44.23) exhibited the highest reporting odds of amenorrhea, whereas norethisterone showed increased reporting odds for ovarian atrophy (ROR = 12.49) and failure (ROR = 8.66) (Table 4).

**Table 4:** Reporting odds ratios of selected COCs for ovarian-function-related preferred terms

Type of PT	Overall COCs		Levonorgestrel		Norethisterone		Desogestrel		Drospirenone		Cyproterone acetate	
	*N*	ROR (95%CI)	*N*	ROR (95%CI)	*N*	ROR (95%CI)	*N*	ROR (95%CI)	*N*	ROR (95%CI)	*N*	ROR (95%CI)
Amenorrhoea	3346	21.59 (20.66–22.57)	2524	33.57 (32.03–35.19)	30	4.62 (3.23–6.61)	41	10.96 (8.06–14.91)	457	44.23 (40.23–48.63)	294	4.82 (4.29–5.42)
Ovarian disorder	21	2.26 (1.46–3.51)	17	3.61 (2.22–5.85)	1	NA	0	NA	1	NA	2	NA
Premature menopause	37	3.80 (2.72–5.31)	32	6.45 (4.51–9.24)	5	6.32 (2.62–15.25)	0	NA	0	NA	0	NA
Ovarian failure	9	1.39 (0.72–2.69)	3	0.98 (0.31–3.06)	6	8.66 (3.86–19.42)	0	NA	0	NA	0	NA
Antral follicle count low	2	NA	2	NA	0	NA	0	NA	0	NA	0	NA
Ovarian atrophy	8	8.39 (3.67–19.17)	2	NA	6	12.49 (5.04–30.96)	0	NA	0	NA	0	NA
Diminished ovarian reserve	1	NA	1	NA	0	NA	0	NA	0	NA	0	NA

Note: N indicates the number of adverse event reports for each preferred term. Reporting odds ratios (RORs) were calculated using all other drugs in FAERS as the comparator group. NA indicates that the ROR was not calculated because the number of reports was fewer than three or the estimate was not applicable. COC, combined oral contraceptive; FAERS, FDA Adverse Event Reporting System; PT, preferred term; ROR, reporting odds ratio

### Network toxicology analysis

#### Effects of EE combined with progestin on DOR

**Fig. 2:**
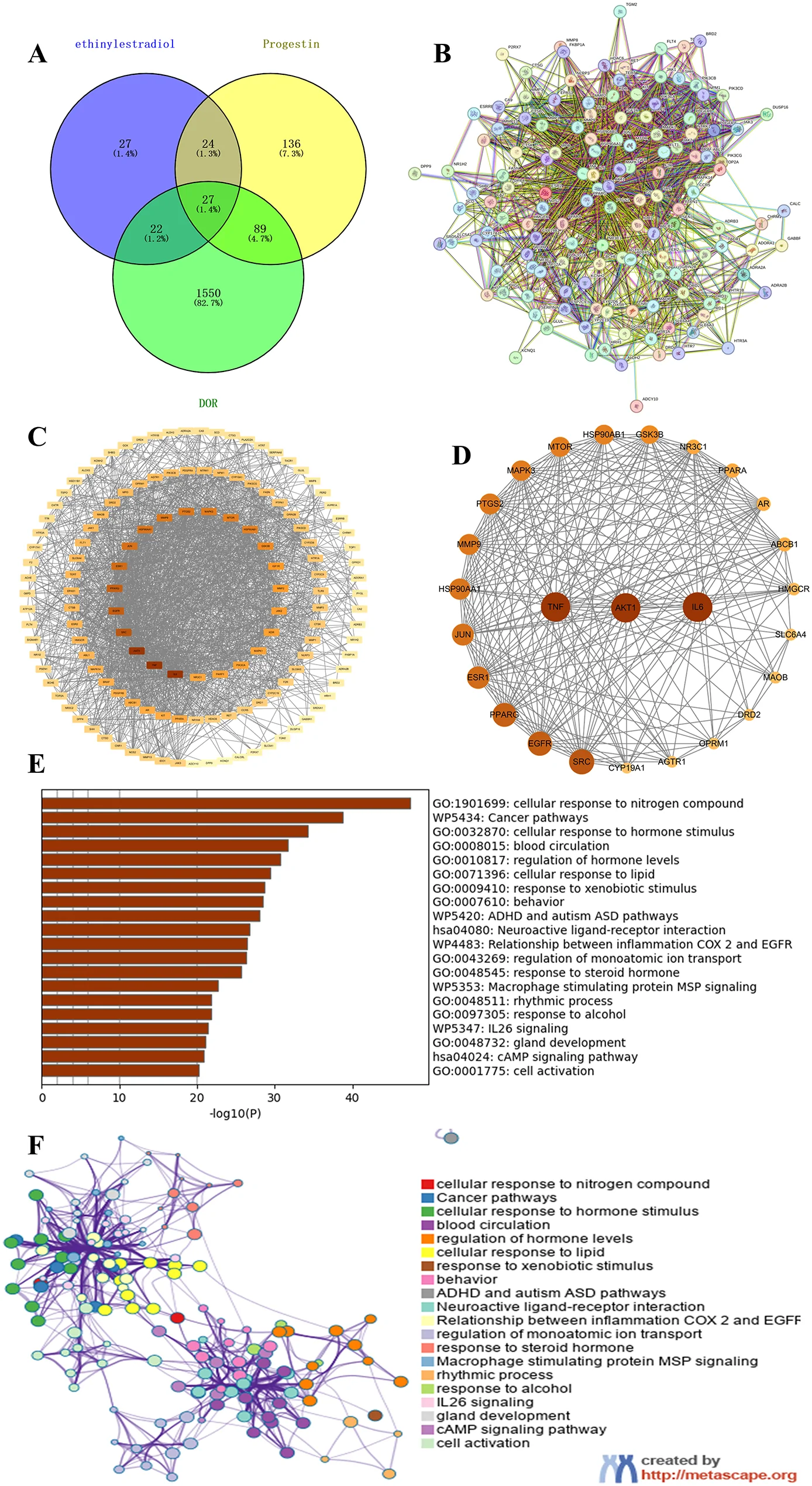
Association analysis between EE combined with PROG‌ and DOR. (**A**) Venn diagram of EE, PROG targets and DOR-related targets; (**B**) PPI network of the intersection targets through STRING database; (**C**) PPI network of the intersection targets through Cytoscape 3.10.3; (**D**) Core targets through CentiScaPe 2.2 plugin; (**E**) KEGG pathway enrichment analysis of the identified targets. Pathways are ranked by their statistical significance, represented as –log10(p-value); (**F**) Illustrates the distribution of KEGG pathways associated with each target

The targets of EE and five clinically common progestins were predicted using the SwissTargetPrediction and DrugBank databases. Following merging and deduplication, 326 targets associated with DOR were identified. Gene targets related to DOR were retrieved from the GeneCards, OMIM, and DisGeNET databases, resulting in 1,688 relevant targets after merging, deduplication, and filtering. A Venn diagram was constructed using the Venny 2.1 platform to compare drug and gene targets, identifying 138 overlapping targets as drug-adverse reaction-related targets (Fig. 2A). These results indicate that these targets may serve as potential mediators of DOR-associated toxic adverse reactions induced by EE in combination with the five clinically common progestins.

A total of 138 targets were imported into the STRING database to construct a PPI network. Database parameters were configured as “Multiple proteins,” with the organism specified as “Homo sapiens” for the query. The resulting target PPI network was exported (Fig. 2B) and visualized using Cytoscape version 3.10.3 (Fig. 2C). These findings provided predictive insights into the intricate interactions between EE in combination with progestin and the pathology of DOR.

The PPI network showed how the proteins in the network interacted with each other. To further explore potential mechanisms underlying DOR-related adverse reaction toxicity induced by the combination of EE and progestin, we employed the CentiScaPe 2.2 plugin within Cytoscape version 3.10.3 to simultaneously identify multiple proteins within the network. The key nodes were discerned through a comprehensive analysis of three parameters—degree, betweenness, and closeness—computed using the plugin (Fig. 2D). These key nodes potentially were ranked according to the degree of prioritization of the criticality of nodes implicated in DOR associated with EE combined with progestin. The top three ranked targets predicted were TNF, AKT1, and IL-6.

Using the Metascape platform, pathway enrichment analysis was performed on 138 adverse drug reaction targets. The results indicated that the cellular response to the nitrogen compound pathway, cancer pathways, and cellular response to hormone stimuli were ranked in the top three pathways, suggesting that these three pathways may act as critical mediators of drug-induced adverse reactions (Fig. 2E, F). These findings support the potential pathways through which EE combined with progestin may contribute to DOR, thereby highlighting predicted key targets and pathways implicated in drug-mediated adverse reactions.

#### Effects of EE combined with progestin on IFA

**Fig. 3:**
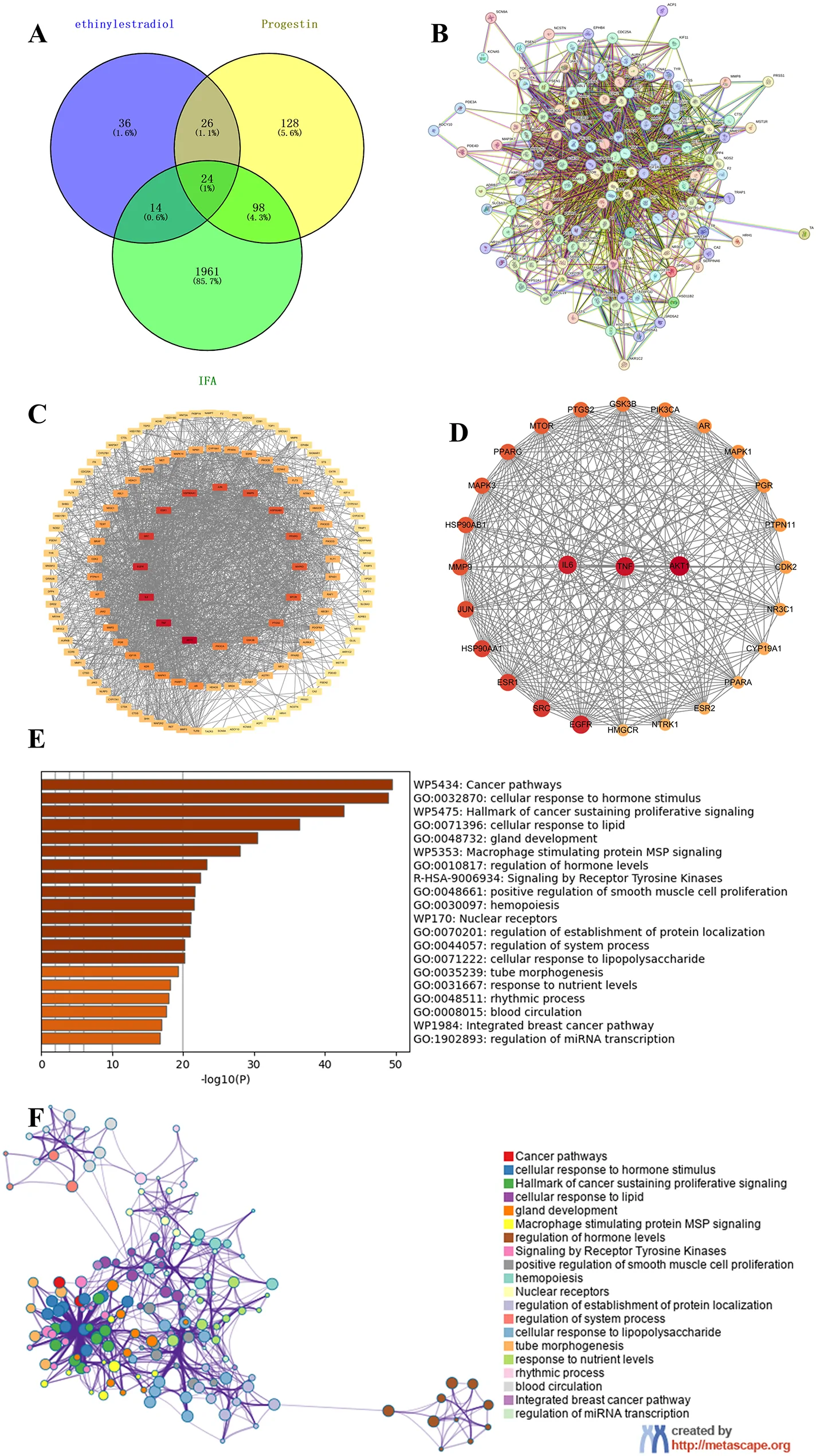
Association analysis between EE‌ combined with PROG‌ and IFA. (**A**) Venn diagram of EE, PROG targets and IFA-related targets; (**B**) PPI network of the intersection targets through STRING database; (**C**) PPI network of the intersection targets through Cytoscape 3.10.3; (**D**) Core targets through CentiScaPe 2.2 plugin; (**E**) KEGG pathway enrichment analysis of the identified targets. Pathways are ranked by their statistical significance, represented as –log10(p-value); (**F**) Illustrates the distribution of KEGG pathways associated with each target

Following the same methodology, a total of 2,097 gene targets associated with IFA were obtained from established databases, yielding 136 overlapping targets with EE combined with progestin after standard processing, as visualized in a Venn diagram (Fig. 3A); these targets represent potential mediators of IFA-associated toxic adverse reactions induced by EE in combination with the five clinically common progestins. A PPI network was constructed using the STRING database (Fig. 3B) and visualized in Cytoscape version 3.10.3 (Fig. 3C) to predict potential interactions between EE-progestin and IFA-related pathology. Core nodes were identified via the CentiScaPe 2.2 plugin through centrality analysis (Fig. 3D), ranking TNF, AKT1, and IL-6 as the top three predicted key targets potentially implicated in these adverse reactions. Pathway enrichment analysis using Metascape on the 136 targets revealed cancer pathways, cellular response to hormone stimulus, and sustained proliferative signaling as the primary mediators of drug-induced toxicity (Figs. 3E, F), thereby suggesting the key pathways and targets through which EE combined with progestin may contribute to IFA.

#### Effects of EE combined with progestin on OD

**Fig. 4:**
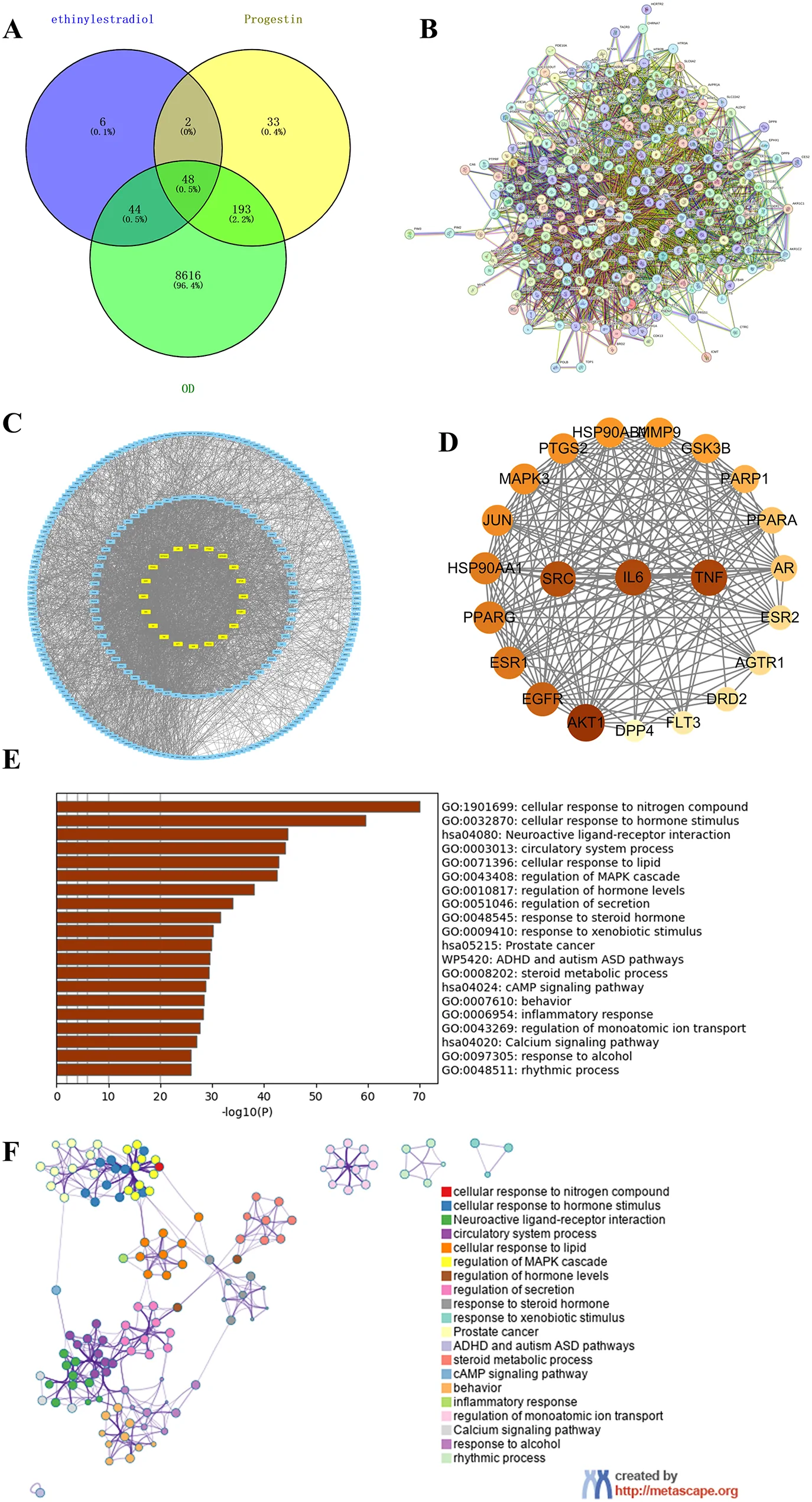
Association analysis between EE combined with PROG‌ and OD. (**A**) Venn diagram of EE, PROG targets and OD-related targets; (**B**) PPI network of the intersection targets through STRING database; (**C**) PPI network of the intersection targets through Cytoscape 3.10.3; (**D**) Core targets through CentiScaPe 2.2 plugin; (**E**) KEGG pathway enrichment analysis of the identified targets. Pathways are ranked by their statistical significance, represented as –log10(p-value); (**F**) Illustrates the distribution of KEGG pathways associated with each target

Similarly, a total of 8,901 gene targets linked to OD were obtained from established databases, yielding 285 overlapping targets with EE combined with progestin after standard processing, as depicted in a Venn diagram (Fig. 4A); these targets likely mediate OD-associated toxic adverse reactions triggered by EE in combination with the five clinically common progestins. A PPI network was built via the STRING database (Fig. 4B) and rendered in Cytoscape version 3.10.3 (Fig. 4C) to model predicted interactions between EE-progestin and OD-related pathology. Key nodes were identified using the CentiScaPe 2.2 plugin through centrality analysis (Fig. 4D), prioritizing TNF, SRC, and IL-6 as top predicted targets in these adverse effects. Pathway enrichment analysis via Metascape on the 285 targets highlighted cellular responses to nitrogen compounds and hormone stimuli, along with the neuroactive ligand-receptor interaction (NLRRI) pathway, as principal drivers of drug-induced toxicity (Figs. 4E, F), thus providing computational insights into the core mechanisms by which EE combined with progestin may promote OD.

#### Effects of EE combined with progestin on OF

**Fig. 5:**
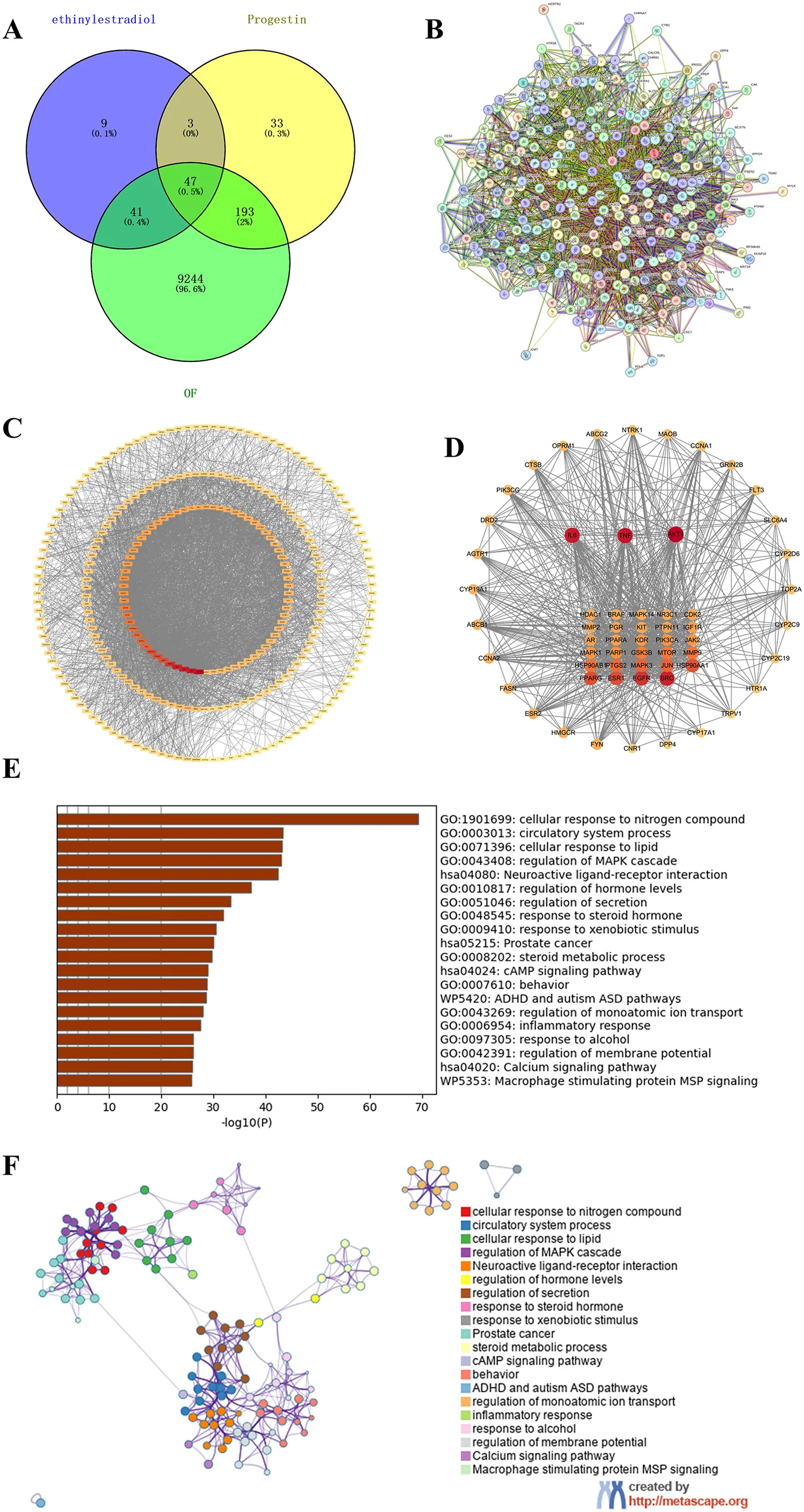
Association analysis between EE combined with PROG‌ and OF. (**A**) Venn diagram of EE, PROG targets and OF-related targets; (**B**) PPI network of the intersection targets through STRING database; (**C**) PPI network of the intersection targets through Cytoscape 3.10.3; (**D**) Core targets through CentiScaPe 2.2 plugin; (**E**) KEGG pathway enrichment analysis of the identified targets. Pathways are ranked by their statistical significance, represented as –log10(p-value); (**F**) Illustrates the distribution of KEGG pathways associated with each target

In a similar fashion, a total of 9,525 gene targets associated with OF were obtained from established databases, yielding 281 overlapping targets with EE combined with progestin after standard processing, as shown in a Venn diagram (Fig. 5A); these targets may serve as potential mediators of OF-associated toxic adverse reactions elicited by EE in combination with the five clinically common progestins. A PPI network was constructed via the STRING database (Fig. 5B) and visualized in Cytoscape version 3.10.3 (Fig. 5C) to predict interactions between EE-progestin and OF-related pathology. Key nodes were identified using the CentiScaPe 2.2 plugin through centrality analysis (Fig. 5D), ranking TNF, AKT1, and IL-6 as the top three predicted targets potentially implicated in these adverse reactions. Pathway enrichment analysis via Metascape on the 281 targets indicated cellular responses to nitrogen compounds, circulatory system processes, and lipids as the primary pathways mediating drug-induced toxicity (Fig. 5E, F), thereby identifying potential core targets and mechanisms by which EE combined with progestin may promote OF.

#### Effects of EE combined with progestin on PM

**Fig. 6:**
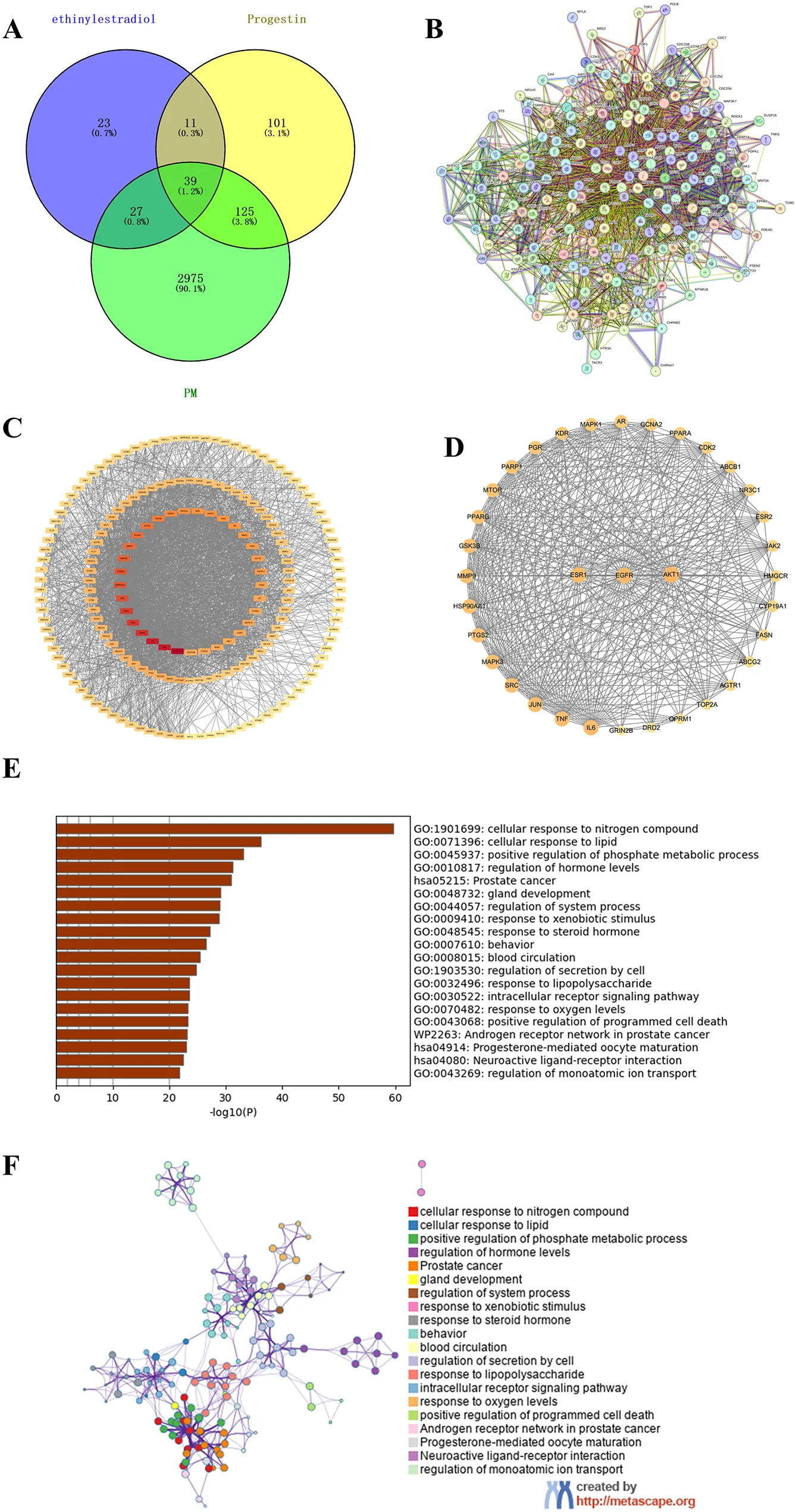
Association analysis between EE‌ combined with PROG‌ and PM. (**A**) Venn diagram of EE, PROG targets and PM-related targets; (**B**) PPI network of the intersection targets through STRING database; (**C**) PPI network of the intersection targets through Cytoscape 3.10.3; (**D**) Core targets through CentiScaPe 2.2 plugin; (**E**) KEGG pathway enrichment analysis of the identified targets. Pathways are ranked by their statistical significance, represented as –log10(p-value); (**F**) Illustrates the distribution of KEGG pathways associated with each target

Likewise, a total of 3,166 gene targets associated with PM were obtained from established databases, yielding 191 overlapping targets with EE combined with progestin after standard processing, as illustrated in a Venn diagram (Fig. 6A); these targets may act as potential mediators of PM-associated toxic adverse reactions elicited by EE in combination with the five clinically common progestins. A PPI network was constructed via the STRING database (Fig. 6B) and visualized in Cytoscape version 3.10.3 (Fig. 6C) to predict interactions between EE-progestin and PM-related pathology. Key nodes were identified using the CentiScaPe 2.2 plugin through centrality analysis (Fig. 6D), ranking ESR1, EGFR, and AKT1 as the top three predicted targets potentially implicated in these adverse reactions. Pathway enrichment analysis via Metascape on the 191 targets revealed cellular responses to nitrogen compounds and lipids, along with positive regulation of phosphate metabolic processes, as the primary pathways mediating drug-induced toxicity (Figs. 6E, F), thereby highlighting the predicted core targets and mechanisms by which EE combined with progestin may be associated with PM.

### Putative metformin-associated modulatory mechanisms

**Fig. 7:**
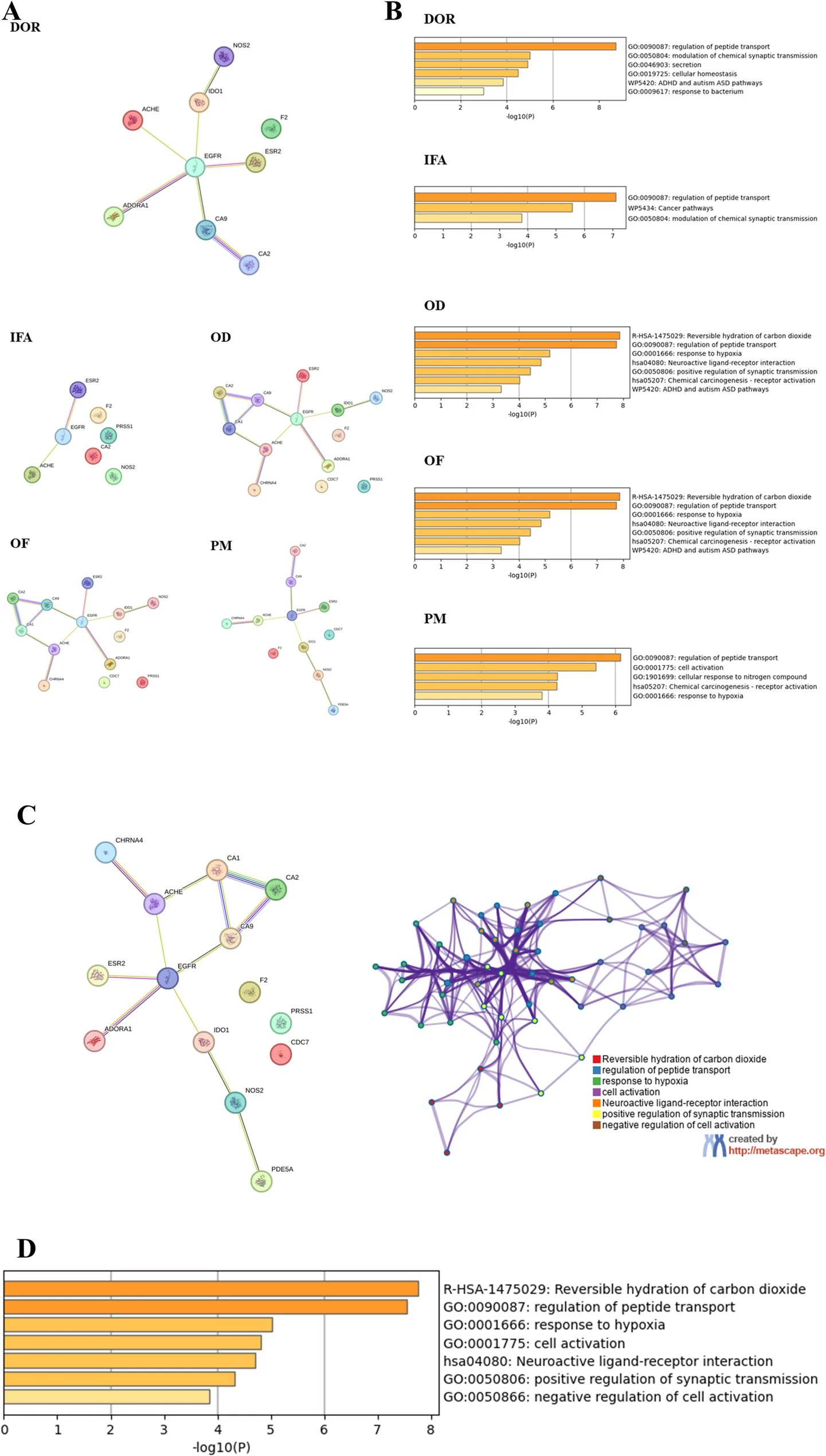
Association analysis of metformin regulation of adverse drug reactions in combined use of EE and PROG. (**A**) The intersection genes of metformin and five adverse reactions caused by EE and PROG drug combination; (**B**) Pathway enrichment of metformin intersecting target genes in five adverse reactions; (**C**) Core targets of the intersection targets of metformin; (**D**) KEGG analysis of core targets

Our analyses explored putative mechanisms underlying the five prevalent adverse reactions associated with EE combined with progestins. Given the clinical observation that documented metformin use was associated with a lower risk of broader ovarian function decline in the cohort, we further explored putative metformin-associated modulatory mechanisms using in silico analyses. The SwissTargetPrediction and DrugBank databases were used to find metformin targets, and Venny 2.1 was used to find possible AE-mitigating targets (Fig. 7A). These intersecting targets underwent pathway enrichment analysis to explore metformin’s potential attenuation of drug-induced adverse reactions (Fig. 7B). After merging and deduplication of metformin-related targets, the hypothesized modulatory effects were linked to 14 genes, including CA2, CA9, ADORA1, and NOS1. Enrichment analysis of these 14 targets revealed associations with pathways like reversible hydration of carbon dioxide, peptide transport regulation, and hypoxia response (Fig. 7C), with the NLRRI pathway showing substantial overlap in most adverse reaction-related enrichments. These results suggest that metformin’s regulation of EE-progestin-induced adverse reactions is tied to the NLRRI pathway, where metformin is predicted to target ADORA1, CA2, NOS2, F2, PRSS1, and CHRNA4, which are implicated.

### AlphaFold 3-based analysis of metformin

Co-folded structures were constructed using AlphaFold3 for all five drug combinations and six targets, followed by interaction analysis (Figures S2–S25). Target comparisons between the five progestins’ AE-associated targets and metformin’s revealed the highest overlaps with DRSP. Given that cyproterone and DRSP are the most common COCs for PCOS treatment in clinical practice, we selected DRSP, EE, and relevant targets. AlphaFold3 predicted the co-folded structures of these drug-target complexes, with accuracy evaluated via ipTM and pTM values. ChimeraX and MOE software were used to visualize and analyze predictive outcomes for ligand-receptor interactions (Fig. 8). The ipTM and pTM values were both above 0.6 and 0.8, respectively, which supported the reliability of the predicted models.

**Fig. 8:**
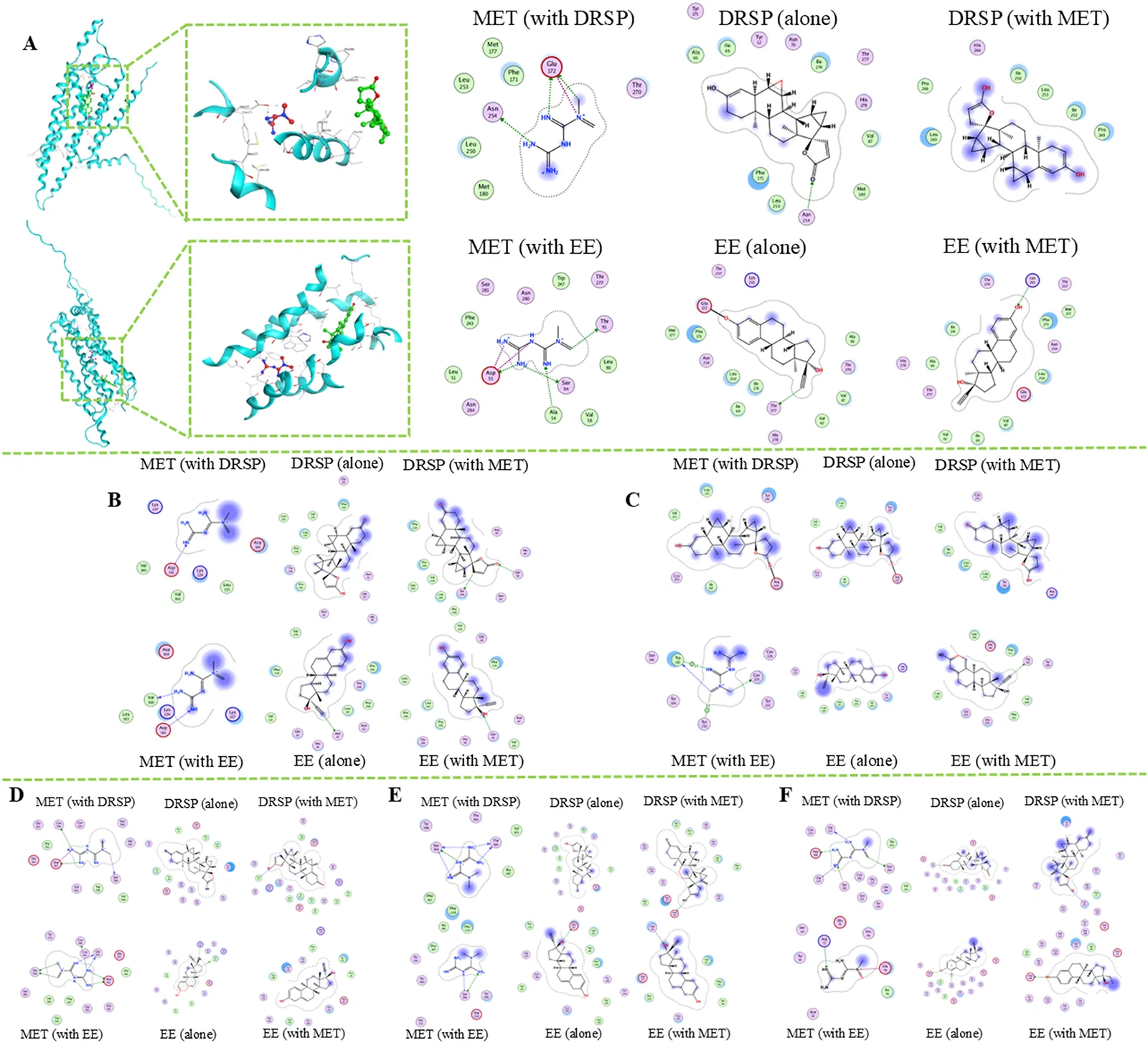
Predicted interactions among metformin, DFRSP, EE, and six putative adverse-reaction-related targets based on AlphaFold 3 co-folded structure prediction. (**A**) The interaction relationship between metformin, EE and DRSP on the ADORA1 target; (**B**) The interaction relationship between metformin, EE and DRSP on the CA2 target; (**C**) The interaction relationship between metformin, EE and DRSP on the NOS2 target; (**D**) The interaction relationship between metformin, EE and DRSP on the F2 target; (**E**) The interaction relationship between metformin, EE and DRSP on the PRSS1; (**F**) The interaction relationship between metformin, EE and DRSP on the CHRNA4 target

MOE-based affinity and interaction analyses showed that DRSP and EE bind to the six targets, forming hydrogen bonds and electron exchanges with proximal binding-pocket amino acids. This implies potential interactions with AE targets that are part of the NLRRI pathway, which may contribute to the observed adverse reactions upon binding. AlphaFold3-based co-folding analysis further indicated that metformin potentially modulates DRSP-EE interactions at the six targets via conformational changes, competitive inhibition, and altered intermolecular forces, suggesting a hypothetical mechanism for regulating adverse reactions through pocket binding associations.

For the ADORA1 target (Fig. 8A), metformin binding was predicted to alter DRSP’s binding angle in the pocket, affecting its interaction with Asn254, and partially overlapped EE’s pocket, modifying EE’s dynamics with Glu172. These changes suggest a possible role for metformin’s regulation of drug-target interactions, reducing AE’s impact on ADORA1. Metformin exerted similar effects on other AE-associated targets by altering binding angles, interaction dynamics, hydrogen bond formation, and pocket positions (Figs. 8B–F). Thus, we hypothesize that it modulates medication-induced AEs by influencing interactions with six key NLRRI pathway targets; however, future wet-lab validation is required to establish definitive causality.

### MD-based evaluation of DRSP /EE–target binding stability and metformin-induced modulation

**Fig. 9:**
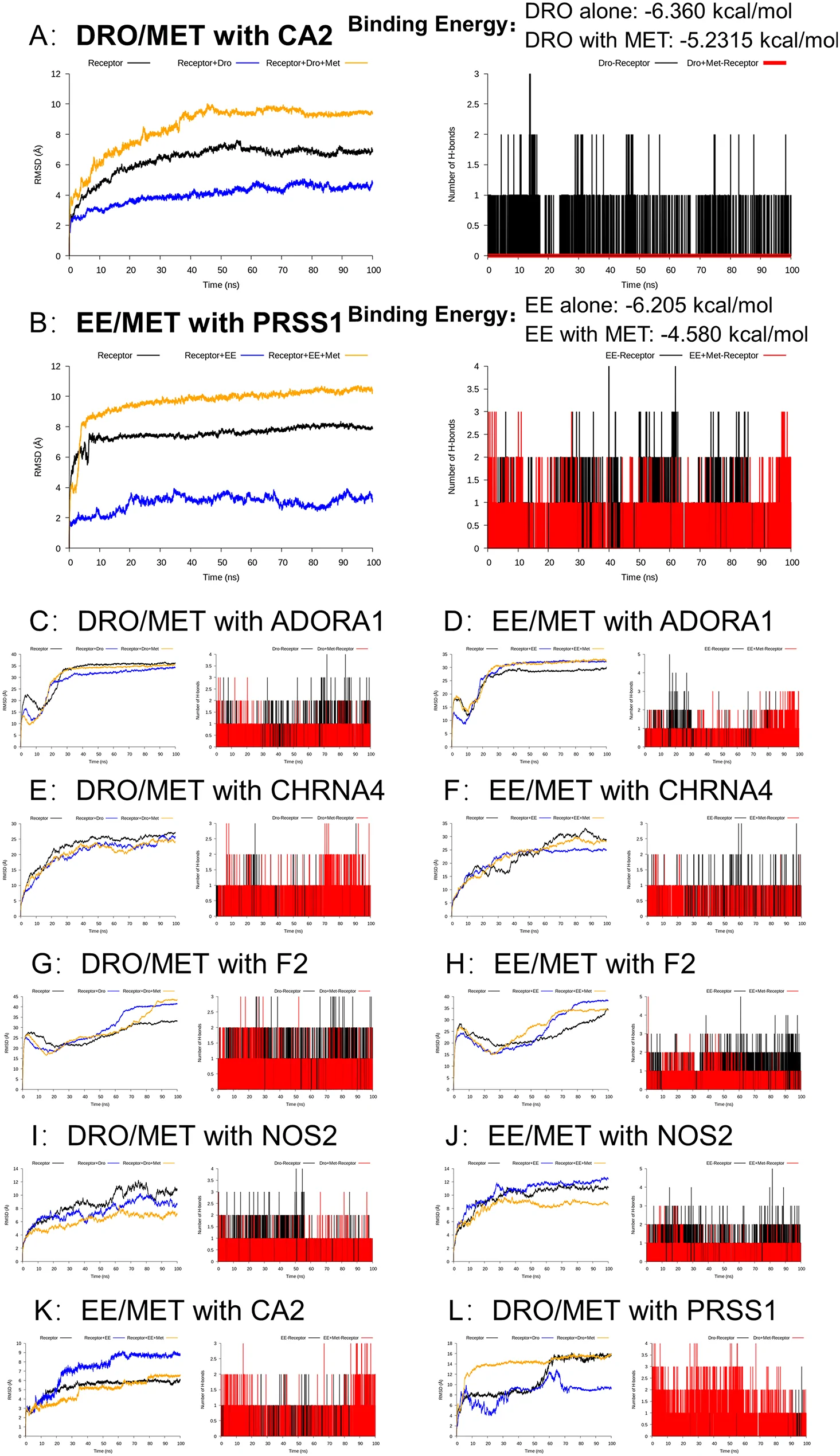
MD simulation of the interactions of DRSP and EE, alone or combined with metformin, with six PCOS-related targets. (**A**) RMSD of CA2 and the number of hydrogen bonds between CA2 and DRSP under DRSP alone and DRSP combined with metformin. (**B**) RMSD of PRSS1 and the number of hydrogen bonds between PRSS1 and EE under EE alone and EE combined with metformin. (**C**) RMSD of ADORA1 and the number of hydrogen bonds between ADORA1 and DRSP under DRSP alone and DRSP combined with metformin. (**D**) RMSD of ADORA1 and the number of hydrogen bonds between ADORA1 and EE under EE alone and EE combined with metformin. (**E**) RMSD of CHRNA4 and the number of hydrogen bonds between CHRNA4 and DRSP under DRSP alone and DRSP combined with metformin. (**F**) RMSD of CHRNA4 and the number of hydrogen bonds between CHRNA4 and EE under EE alone and EE combined with metformin. (**G**) RMSD of F2 and the number of hydrogen bonds between F2 and DRSP under DRSP alone and DRSP combined with metformin. (**H**) RMSD of F2 and the number of hydrogen bonds between F2 and EE under EE alone and EE combined with metformin. (**I**) RMSD of NOS2 and the number of hydrogen bonds between NOS2 and DRSP under DRSP alone and DRSP combined with metformin. (**J**) RMSD of NOS2 and the number of hydrogen bonds between NOS2 and EE under EE alone and EE combined with metformin. (**K**) RMSD of CA2 and the number of hydrogen bonds between CA2 and EE under EE alone and EE combined with metformin. (**L**) RMSD of PRSS1 and the number of hydrogen bonds between PRSS1 and DRSP under DRSP alone and DRSP combined with metformin

From prior target prediction and network toxicology analyses, six targets computationally linked to DRSP- and EE-induced adverse reactions in PCOS were identified: CA2, PRSS1, ADORA1, CHRNA4, F2, and NOS2. 100-ns MD simulations assessed their binding stability and dynamics with DRSP/metformin and EE/metformin complexes. For most targets, DRSP- or EE-bound systems stayed stable, but RMSD rose markedly with metformin, implying altered binding modes or conformational shifts. This suggests that metformin may alter the stability of selected COC–target complexes, providing a putative mechanism relevant to the observed associations. Responses to DRSP and EE varied by target, with CA2 and PRSS1 exhibiting heightened sensitivity and pronounced RMSD fluctuations (Fig. 9), marking them as potential perturbation-sensitive targets central to these adverse mechanisms.

As shown in Fig. 9A, apo CA2 showed moderate RMSD fluctuations, indicating stable native conformation. DRSP binding alone kept RMSD low (2–4 Å), below apo levels, suggesting a stable complex contributing to DRSP-induced PCOS effects. However, in the DRSP–metformin–CA2 system, RMSD surged to 8–10 Å with high variability, indicating metformin disruption of DRSP–CA2 binding and stability—a potential mechanism counteracting DRSP-associated reactions. DRSP–CA2 hydrogen bonds persisted with minimal fluctuation, indicating computational stability, whereas those in the ternary complex were unstable and often broken, aligning with elevated RMSD.

Similarly, EE introduction reduced PRSS1 RMSD fluctuations (Fig. 9B), forming a stable EE–PRSS1 complex. In contrast, the EE–metformin–PRSS1 system showed substantial RMSD increases, the least stable among PRSS1 configurations. This destabilization implies metformin interference with EE–PRSS1 binding, possibly perturbing active sites or functional loops to attenuate EE-induced PCOS effects, which remains to be experimentally verified.

## Discussion

In this multisource study, integrating a real-world cohort, FAERS pharmacovigilance signals, and network toxicology with molecular modeling, we observed that prolonged COC exposure was associated with increased odds of severe ovarian function decline (POI + POF) in women with PCOS, while documented metformin use was associated with lower odds of the broader ovarian function decline outcome. Computational predictions suggested that COCs-related ovarian toxicity was associated with inflammatory, oxidative stress, and hormone-response pathways, with principal targets potentially including TNF, AKT1, IL-6, SRC, ESR1, and EGFR, and the hypothesized involvement of the NLRRI pathway. Based on our molecular modeling, we hypothesize that metformin could mitigate these effects through competitive binding and conformational modulation of drug–target complexes.

Our retrospective cohort analysis utilizing multiple imputation revealed a significant association between prolonged COCs use (≥ 2 years) and the risk of ovarian function decline in patients with PCOS. Among 336 women of reproductive age with early-diagnosed PCOS, the incidence of secondary ovarian function decline among COCs users was 20.4%, which markedly exceeded that observed in non-users. Following multivariable logistic regression adjustment for confounding factors, prolonged COCs use remained robustly associated with an elevated risk of severe ovarian function decline (POI and POF); however, this association was no longer significant for the broader definition (encompassing pre-POI). These findings suggest that prolonged COC use may be more strongly associated with clinically overt ovarian function decline than with earlier or prodromal changes. Conversely, the attenuation of statistical significance for the broader outcome in the fully adjusted model indicates that COCs may not independently influence the initial stages of ovarian reserve diminution—which may be more heavily confounded by underlying metabolic profiles, yet could promote progression to clinically overt insufficiency.

Furthermore, our pharmacovigilance analysis of the FAERS database reinforced this association, providing real-world evidence to complement the cohort findings. Across 2,415 reports of COCs-related ovarian function decline from 2014 to 2024, these events predominantly occurred in women of reproductive age, primarily manifesting as AEs such as amenorrhea, PM, and ovarian atrophy. Subgroup analyses revealed significant risk variations across different progestin components, with levonorgestrel and DRSP exhibiting the highest risks for amenorrhea, and norethisterone demonstrating prominent risks for OF. Such results may reflect the pro-oxidative effects of progestins (e.g., activation of pro-oxidative enzymes) that counteract the antioxidative actions of estrogens (e.g., inhibition of NADPH oxidase), resulting in dose-dependent toxicity [38]. Although FAERS is susceptible to reporting biases and causal uncertainties (e.g., consumer reports comprising 76.45%, potentially hypersensitive to menstrual irregularities), the alignment with cohort findings furnishes additional caution and supports the risk assessment of COCs in long-term PCOS management [16, 25, 28]. These clinical observations establish a foundation for subsequent mechanistic explorations, underscoring the necessity to re-evaluate COCs’ ovarian safety within the PCOS-specific context to avert potential “double-edged sword” effects [5].

The documented history of metformin use in our cohort was associated with lower odds of broad ovarian-function decline, a biologically plausible finding that is consistent with prior mechanistic studies suggesting that metformin may attenuate ovarian oxidative and inflammatory pathways [18–20]. According to the 2023 international PCOS guideline, metformin is recommended for adults with BMI ≥ 25 kg/m² for metabolic indications, with gradual titration in 500-mg increments and the use of extended-release formulations to improve tolerability—features that highlight the importance of adequate and sustained exposure [39]. Recent reviews also emphasize that improvements in insulin resistance and hyperandrogenism may support healthier follicular dynamics, while gastrointestinal side effects can limit adherence and thereby modulate treatment effectiveness [40]. In addition, a 2024 meta-analysis reported that metformin is associated with reductions in AMH that appear sensitive to treatment dose and duration, and noted that at least 12 weeks of therapy are generally required to observe meaningful changes, suggesting that treatment duration is a relevant determinant of ovarian response [41]. Collectively, these data point out the need for prospective studies that capture detailed information on metformin dose, duration, and adherence to clarify exposure–response relationships and mechanisms underlying potential ovarian protection.

Building on COC-related ovarian-function signals and the metformin-associated protective pattern observed in our cohort, network toxicology analysis offers preliminary mechanistic insights into their molecular interplay, potentially linking observed clinical risks to biological pathways. The ovarian toxicity target set was constructed using high ROR signals from FAERS (e.g., amenorrhea) to guide keyword selection, intersecting these genes with COCs targets to yield 138 shared ones predicted to map to outcomes like DOR and IFA. The PPI network highlighted high-centrality predicted core targets, including TNF, AKT1, IL-6, SRC, ESR1, EGFR, and ADORA1, which are potentially central to inflammatory cascades, oxidative stress, and hormone signaling—aligning with PCOS pathophysiology like chronic inflammation and granulosa cell apoptosis [42, 43]. A KEGG enrichment analysis of five adverse reaction intersection genes evaluated pathway convergence for core targets TNF, AKT1, and IL-6, revealing substantial overlaps, especially in inflammation pathways. A summary figure (Supplementary Figure S1) visually presents the shared architecture. Enrichment analysis showed significant over-representation of the NLRRI pathway (*P* < 0.001), alongside inflammatory and oxidative stress modules. This suggests that COCs use may upregulate pro-inflammatory cytokines (e.g., TNF-α, IL-6) and potentially activate AKT1-mediated follicular atresia, which might exacerbate ovarian atrophy—consistent with FAERS reports (e.g., PM in 37 cases) and cohort outcomes (e.g., 20.4% incidence in COCs users) [25–28]. To further interrogate these interactions, we conducted molecular docking and MD simulations, using AlphaFold 3 to generate initial models for five COCs combinations and six key targets; docking simulations predicted stable binding of COCs to these proteins, potentially promoting pro-oxidative effects that counteract estrogen’s antioxidative actions (e.g., NADPH oxidase inhibition) [31, 32, 44, 45]. MD simulations further supported the potential of metformin’s destabilizing influence on COCs–target complexes, with elevated RMSD in ternary systems (e.g., DRSP–metformin–CA2) indicating simulated competitive disruption of binding pockets, potentially via AMPK-mediated shifts in NLRRI signaling. In contrast, metformin was predicted to exhibit competitive binding (e.g., to AKT1 and IL-6) with lower affinity, inducing conformational changes that reduced complex stability, as evidenced by higher RMSF fluctuations and lower binding energies in silico. This supports a computational model in which metformin modulates NLRRI signaling via AMPK activation and mTOR reprogramming [18], potentially attenuating ROS/inflammation and offering a mechanistic hypothesis for the lower risk of ovarian function decline associated with metformin use observed in our clinical cohort. However, these predictions remain hypothesis-generating and require wet-lab validation to establish causality.

The findings indicate important clinical implications, which emphasize the requirement for personalized and risk-stratified management of women with PCOS. The administration of long-term COCs should be tailored to individual factors, including BMI, AMH levels, fertility objectives, and anticipated treatment duration, in accordance with international evidence-based guidelines to maintain a balance between efficacy and ovarian safety. Metformin use may be regarded not only for metabolic purposes but also for its potential ovarian-protective associations during expected prolonged COCs use, while refraining from causal overinterpretation until randomized evidence is available. From a translational perspective, these signals are currently hypothesis-generating rather than practice-changing. They require validation in prospective PCOS cohorts, real-world registries with standardized definitions of ovarian function decline and adjudicated POI outcomes, as well as pragmatic trials comparing COCs ± metformin to identify benefiting subgroups and inform risk-stratified contraceptive counseling.

This study presents some limitations that should be acknowledged. The retrospective cohort design limits causal inference, relying on existing data without fully establishing temporality or directionality between COCs exposure and ovarian function decline, which may over- or underestimate associations. Although multivariable models adjusted for key confounders, inherent biases persist, including selection, recall, unmeasured confounding, and incomplete follow-up. The sample size of 336 patients supports preliminary observations but may limit generalizability across diverse racial or socioeconomic groups. Second, the FAERS pharmacovigilance analysis has inherent limitations: FAERS data are subject to underreporting, stimulated reporting, selective reporting, missing information, and lack of denominator data; therefore, incidence rates and causal risks cannot be estimated. We mitigated random noise with a threshold of at least three cases for ROR calculations and addressed duplication bias via data cleaning to remove duplicates before analysis (as detailed in Methods). Most critically, indication bias (confounding by indication) is a major challenge, as PCOS inherently raises OD risk, potentially generating signals from prescribing to high-risk populations. Although disproportionality analysis cannot eliminate this bias, we contextualized findings by integrating with a PCOS-specific clinical cohort, offering stronger evidence via multi-source triangulation than standalone FAERS use. Nonetheless, ROR signals potential associations, not causal risks, requiring prospective validation. Finally, network toxicology and molecular modeling provide mechanistic insights but depend on database predictions that may overlook in vivo complexities or variability, necessitating wet-lab validation. These limitations necessitate cautious interpretation and prospective randomized trials to validate findings, address unmeasured variables, and improve global prevention and care for reproductive endocrine disorders through comprehensive studies.

## Conclusion

This study represents the first integration of multisource data to systematically characterize clinical and pharmacovigilance signals and explore predicted molecular mechanisms of long-term COCs therapy on ovarian function in patients with PCOS. Predicted involvement of inflammatory, oxidative stress, and hormone-response pathways, along with the putative roles of computationally identified core targets such as TNF, AKT1, and IL-6, may help contextualize the observed clinical and pharmacovigilance signals. The findings indicate that COCs use may be associated with dual effects, potentially elevating the risk of secondary POI while concurrently alleviating symptoms. These results highlight the need for a careful evaluation of immediate advantages versus long-term ovarian safeguarding in PCOS management and suggest that a history of metformin use may be associated with lower odds of broader ovarian-function decline, potentially in relation to predicted modulation of the NLRRI pathway. Given the observational and predictive nature of our study, future prospective or randomized investigations, alongside experimental wet-lab validation, are warranted to confirm these clinical associations and hypothesized protective mechanisms, thereby optimizing individualized management of PCOS.

## Supplementary Information

Below is the link to the electronic supplementary material.

Supplementary Material 1

Supplementary Material 2

## Data Availability

The data supporting the findings of this study are available from the corresponding author upon reasonable request.

## Abbreviations

AEs: Adverse events
AMH: Anti-Müllerian hormone
BMI: Body mass index
CI: Confidence interval
COCs: Combined oral contraceptives
DOR: Diminished ovarian reserve
DRSP: Drospirenone
EE: Ethinylestradiol
FDA: Food and Drug Administration
FAERS: FDA Adverse Event Reporting System
FSH: Follicle-stimulating hormone
IFA: Increased follicular atresia
KEGG: Kyoto Encyclopedia of Genes and Genomes
MD: Molecular dynamics
NLRRI: Neuroactive ligand-receptor interaction
NOR: Normal ovarian reserve
OD: Ovarian dysfunction
OF: Ovarian failure
PCOS: Polycystic ovarian syndrome
PM: Premature menopause
POF: Premature ovarian failure
POI: Premature ovarian insufficiency
PPI: Protein–protein interaction
PT: Preferred terms
