# Supplementary material for: Long-term ovarian function risks associated with combined oral contraceptive administration in polycystic ovary syndrome: multisource data integration and protective mechanisms of metformin
Source: J Transl Med. 2026 Jun 15;24:1087. doi: 10.1186/s12967-026-08429-y (PMC13504797; doi:10.1186/s12967-026-08429-y)
Supplement: Supplementary file 1 [file 12967_2026_8429_MOESM1_ESM.docx]

Figure S1 ‌5 adverse reaction intersection genes and KEGG analysis results

Figure S2 The co-crystallization structure of drospirenone and ADORA1 predicted based on AlphaFold3

Figure S3 The co-crystallization structure of drospirenone, Metformin and ADORA1 predicted based on AlphaFold3

Figure S4 The co-crystallization structure of ethynylestradiol and ADORA1 predicted based on AlphaFold3

Figure S5 The co-crystallization structure of ethynylestradiol, Metformin and ADORA1 predicted based on AlphaFold3

Figure S6 The co-crystallization structure of drospirenone and CA2 predicted based on AlphaFold3

Figure S7 The co-crystallization structure of drospirenone, Metformin and CA2 predicted based on AlphaFold3

Figure S8 The co-crystallization structure of ethynylestradiol and CA2 predicted based on AlphaFold3

Figure S9 The co-crystallization structure of ethynylestradiol, Metformin and CA2 predicted based on AlphaFold3

Figure S10 The co-crystallization structure of drospirenone and CHRNA4 predicted based on AlphaFold3

Figure S11 The co-crystallization structure of drospirenone, Metformin and CHRNA4 predicted based on AlphaFold3

Figure S12 The co-crystallization structure of ethynylestradiol and CHRNA4 predicted based on AlphaFold3

Figure S13 The co-crystallization structure of ethynylestradiol, Metformin and CHRNA4 predicted based on AlphaFold3

Figure S14 The co-crystallization structure of drospirenone and F2 predicted based on AlphaFold3

Figure S15 The co-crystallization structure of drospirenone, Metformin and F2 predicted based on AlphaFold3

Figure S16 The co-crystallization structure of ethynylestradiol and F2 predicted based on AlphaFold3

Figure S17 The co-crystallization structure of ethynylestradiol, Metformin and F2 predicted based on AlphaFold3

Figure S18 The co-crystallization structure of drospirenone and NOS2 predicted based on AlphaFold3

Figure S19 The co-crystallization structure of drospirenone, Metformin and NOS2 predicted based on AlphaFold3

Figure S20 The co-crystallization structure of ethynylestradiol and NOS2 predicted based on AlphaFold3

Figure S21 The co-crystallization structure of ethynylestradiol, Metformin and NOS2 predicted based on AlphaFold3

Figure S22 The co-crystallization structure of drospirenone and PRSS1 predicted based on AlphaFold3

Figure S23 The co-crystallization structure of drospirenone, Metformin and PRSS1 predicted based on AlphaFold3

Figure S24 The co-crystallization structure of ethynylestradiol and PRSS1 predicted based on AlphaFold3

Figure S25 The co-crystallization structure of ethynylestradiol, Metformin and PRSS1 predicted based on AlphaFold3
