# Supplementary material for: Long-term ovarian function risks associated with combined oral contraceptive administration in polycystic ovary syndrome: multisource data integration and protective mechanisms of metformin
Source: J Transl Med. 2026 Jun 15;24:1087. doi: 10.1186/s12967-026-08429-y (PMC13504797; doi:10.1186/s12967-026-08429-y)
Supplement: Supplementary file 2 [file 12967_2026_8429_MOESM2_ESM.docx]

| Supplementary Table S1  Molecular formula, molecular weight and SMILES structure of EDCs. | | | |
| --- | --- | --- | --- |
| Name | Molecular formulas | Molecular weights (g/mol) | SMILES structures |
| ethinylestradiol | C20H24O2 | 296.4 | C[C@]12CC[C@H]3[C@H]([C@@H]1CC[C@]2(C#C)O)CCC4=C3C=CC(=C4)O |
| cyproterone | C22H27ClO3 | 374.9 | CC(=O)[C@]1(CC[C@@H]2[C@@]1(CC[C@H]3[C@H]2C=C(C4=CC(=O)[C@@H]5C[C@@H]5[C@]34C)Cl)C)O |
| drospirenone | C24H30O3 | 366.5 | C[C@]12CCC(=O)C=C1[C@@H]3C[C@@H]3[C@@H]4[C@@H]2CC[C@]5([C@H]4[C@@H]6C[C@@H]6[C@@]57CCC(=O)O7)C |
| desogestrel | C22H30O | 310.5 | CC[C@]12CC(=C)[C@H]3[C@H]([C@@H]1CC[C@]2(C#C)O)CCC4=CCCC[C@H]34 |
| levonorgestrel | C21H28O2 | 312.4 | CC[C@]12CC[C@H]3[C@H]([C@@H]1CC[C@]2(C#C)O)CCC4=CC(=O)CC[C@H]34 |
| norethisterone | C20H26O2 | 298.4 | C[C@]12CC[C@H]3[C@H]([C@@H]1CC[C@]2(C#C)O)CCC4=CC(=O)CC[C@H]34 |
| Metformin | C4H11N5 | 129.16 | CN(C)C(=N)N=C(N)N |

**Supplementary Table S2. Missingness profile and Little’s MCAR test results.**

**Part A. Missingness profile**

| **Variable** | **Missing type** | **Missing, n** | **Missing, %** |
| --- | --- | --- | --- |
| Height | Random | 12 | 3.57 |
| Weight | Random | 12 | 3.57 |
| Testosterone | Random | 8 | 2.38 |
| AMH | Random | 7 | 2.08 |
| COC discontinuation duration among COC users | Random within COC users | 7 | 4.19 |

**Part B. Little’s MCAR test**

| **Test** | **χ²** | **df** | **P value** | **Missing patterns** |
| --- | --- | --- | --- | --- |
| Little’s MCAR test for four global root variables | 11.542 | 8 | 0.173 | 4 |
| Little’s MCAR test including COC discontinuation duration among COC users | 22.627 | 20 | 0.307 | 7 |

Note: BMI was derived from height and weight and was not treated as an independent missing variable. Little’s MCAR tests did not reject the MCAR assumption. Multiple imputation was performed under the MAR framework using predictive mean matching. AMH, anti-Müllerian hormone; BMI, body mass index; COC, combined oral contraceptive; MCAR, missing completely at random; MAR, missing at random.

**Supplementary Table S3：Available-data univariable logistic regression analyses before MI；**

| Variable | Outcome 1 Univariate logistic OR (95% CI) | P | Outcome 2 Univariate logistic OR (95% CI) | P |
| --- | --- | --- | --- | --- |
| Age at PCOS diagnosis, years | 0.989 (0.879,1.112) | 0.848 | 0.972 (0.905,1.044) | 0.441 |
| Age at COC initiation, years | 1.041 (0.901,1.203) | 0.582 | 1.012 (0.926,1.106) | 0.793 |
| BMI at PCOS diagnosis |  |  |  |  |
| BMI＜25kg/m2 | 1.00 (ref) | - | 1.00 (ref) | - |
| BMI≥25kg/m2 | 1.018 (0.343,3.023) | 0.974 | 1.481 (0.735,2.984) | 0.272 |
| Therapy |  |  |  |  |
| Short-term/never COCs use（＜2 years） | 1.00 (ref) | - | 1.00 (ref) | - |
| Long-term COCs use（≥2 years） | 6.620 (2.006,21.851) | 0.002 | 3.048 (1.204,7.712) | 0.019 |
| Duration of COCs discontinuation |  |  |  |  |
| ＜12 months | 1.00 (ref) | - | 1.00 (ref) | - |
| ≥12 months | 0.464 (0.133,1.621) | 0.229 | 0.954 (0.410,2.220) | 0.913 |
| Metformin use | 0.351 (0.090,1.364) | 0.131 | 0.567 (0.068,4.748) | 0.6 |

Note: These analyses were performed using the available clinical dataset before multiple imputation and are provided as supplementary analyses for transparency. The primary regression analyses are based on pooled multiple-imputation models and are presented in Table 2 of the main manuscript. Outcome 1 was defined as POI + POF; Outcome 2 was defined as pre-POI + POI + POF. Adjusted models were adjusted for calendar time, diabetes, and insulin resistance. COC, combined oral contraceptive; OR, odds ratio; PCOS, polycystic ovary syndrome; POI, premature ovarian insufficiency; POF, premature ovarian failure.

| **Supplementary Table S4. Available-data logistic regression analyses before multiple imputation.** | | | | | | | | |
| --- | --- | --- | --- | --- | --- | --- | --- | --- |
| Variable | Outcome 1 Crude Odds Ratio (95% CI) | P | Outcome 1 Adjusted Odds Ratio (95% CI) * | P | Outcome 2 Crude Odds Ratio (95% CI) | P | Outcome 2 Adjusted Odds Ratio (95% CI) * | P |
| Age at PCOS diagnosis, years, median (quartiles) | 0.975 (0.870,1.092) | 0.658 | 0.912 (0.769,1.080) | 0.286 | 0.964 (0.900,1.034) | 0.309 | 0.938 (0.851,1.034) | 0.199 |
| Age for COCs medication, years, median(quartiles) | 1.035 (0.898,1.192) | 0.639 | 1.055 (0.894,1.244) | 0.528 | 1.007 (0.923,1.099) | 0.868 | 1.012 (0.924,1.109) | 0.8 |
| BMI at PCOS diagnosis, n (%) |  |  |  |  |  |  |  |  |
| BMI＜25kg/m2 | 1.00 (ref) | - | 1.00 (ref) | - | 1.00 (ref) | - | 1.00 (ref) | - |
| BMI≥25kg/m2 | 1.044 (0.354,3.084) | 0.938 | 1.136 (0.287,4.503) | 0.856 | 1.500 (0.747,3.014) | 0.255 | 1.514 (0.659,3.481) | 0.329 |
| Therapy, n (%) |  |  |  |  |  |  |  |  |
| Short-term/never COCs use (＜2 years) | 1.00 (ref) | - | 1.00 (ref) | - | 1.00 (ref) | - | 1.00 (ref) | - |
| Long-term COCs use (≥2 years) | 6.818 (2.159,21.352) | 0.001 | 7.53 (1.770,32.026) | 0.006 | 3.188 (1.292,7.870) | 0.012 | 2.360 (0.849,6.560) | 0.1 |
| Duration of COCs discontinuation, n (%) |  |  |  |  |  |  |  |  |
| ＜12 months | 1.00 (ref) | - | 1.00 (ref) | - | 1.00 (ref) | - | 1.00 (ref) | - |
| ≥12 months | 0.414 (0.120,1.430) | 0.163 | 0.419 (0.105,1.679) | 0.219 | 0.857 (0.373,1.971) | 0.717 | 0.932 (0.382,2.272) | 0.877 |
| Metformin use | 0.400 (0.112,1.434) | 0.160 | 0.160 (0.030,0.869) | 0.034 | 0.707 (0.363,1.377) | 0.308 | 0.361 (0.155,0.838) | 0.018 |

Note: These analyses were performed using the available clinical dataset before multiple imputation and are provided as supplementary analyses for transparency. The primary regression analyses are based on pooled multiple-imputation models and are presented in Table 2 of the main manuscript. Outcome 1 was defined as POI + POF; Outcome 2 was defined as pre-POI + POI + POF. Adjusted models were adjusted for calendar time, diabetes, and insulin resistance. COC, combined oral contraceptive; OR, odds ratio; PCOS, polycystic ovary syndrome; POI, premature ovarian insufficiency; POF, premature ovarian failure.
